# Gut Microbiome Signatures of Aging Associated with Intramuscular Fat Deposition in Tan Sheep

**DOI:** 10.3390/ani16040661

**Published:** 2026-02-19

**Authors:** Xin Yuan, Xuelong Su, Daohua Zhuang, Huitong Zhou, Zecheng Tang, Chenshuo Li, Jiqing Wang, Bingang Shi, Yuzhu Luo, Shaobin Li, Fangfang Zhao

**Affiliations:** 1 Gansu Key Laboratory of Herbivorous Animal Biotechnology, Faculty of Animal Science and Technology, Gansu Agricultural University, Lanzhou 730070, China; yuanxin722722@outlook.com (X.Y.); s2658747838@gmail.com (X.S.); lcs1984580269@outlook.com (C.L.); wangjq@gsau.edu.cn (J.W.); shibg@gsau.edu.cn (B.S.); luoyz@gsau.edu.cn (Y.L.); 2 Kunming Institute of Zoology, Chinese Academy of Sciences, Kunming 650201, China; grasstrong@gmail.com; 3 Gene-Marker Laboratory, Faculty of Agricultural and Life Sciences, Lincoln University, Lincoln 7647, New Zealand; huitong.zhou@lincoln.ac.nz; 4 State Key Laboratory for Conservation and Utilization of Bioresources in Yunnan, School of Life Sciences, Yunnan University, Kunming 650091, China; tangzecheng1999@163.com

**Keywords:** Tan sheep, age-related IMF deposition, lipid levels, lipid-metabolizing enzymes, short-chain fatty acids, gut microbiota

## Abstract

Intramuscular fat (IMF) content determines marbling levels and influences the sensory and edible qualities of livestock meat. Its deposition is influenced by the animal’s age and gut microbial community. This study assessed age-related differences in IMF deposition and shifts in gut microbiota between yearlings (1-year-old) and mature (4-year-old) grazing Tan sheep. Then correlations among these factors were examined to investigate the potential role of gut bacteria in IMF deposition. The results demonstrated that mature sheep exhibited higher IMF content in shoulder and rump muscles (*p* < 0.05), elevated serum lipid levels (*p* < 0.001), and increased lipolytic enzyme abundances in the liver and pancreas (*p* < 0.05), compared with yearlings. In contrast, the concentrations of acetate and propionate in ruminal and colonic contents were lower in mature sheep (*p* < 0.05), despite a higher abundance of lipolytic and synthetic enzymes in colonic content (*p* < 0.05). Gut microbial diversity differed between age groups, particularly in the rumen and colon, with clear shifts in specific bacterial taxa. Correlation analyses revealed that the abundance of *Copromorpha* and *RUG420* in the colon were positively correlated with IMF content in shoulder and rump muscles, and serum lipid levels (including free fatty acids, FFA; low-density lipoprotein, LDL; high-density lipoprotein, HDL; and very-low-density lipoprotein, VLDL), but negatively correlated with propionate content (|*r*| > 0.45, *FDR* < 0.05). Conversely, the abundance of *Cryptobacteroides* in the colon was negatively correlated with IMF content in shoulder muscle (*r* < −0.6, *FDR* < 0.05), and with the levels of triglyceride (TG), LDL, HDL, and VLDL, while showing positive correlations with acetate and propionate contents (*r* > 0.45, *FDR* < 0.05). These findings highlight the potential role of specific colon bacteria (*Copromorpha*, *RUG420*, and *UBA5905*) in IMF deposition, identifying them as candidate bacteria for further investigation regarding their effects on meat quality.

## Simple Summary

Intramuscular fat (IMF) is a key factor in determining meat quality and palatability. Identifying the regulators of IMF deposition is essential for developing strategies to improve meat quality. Previous studies have shown that certain dietary additives and candidate genes promote fat accumulation. This present study highlights the significant influence of age on IMF deposition in Tan sheep. Mature sheep exhibit higher IMF content in the shoulder and rump muscles, elevated serum lipid levels, and increased concentrations of lipolytic enzymes in the liver and pancreas compared to yearlings. These age-related changes were associated with shifts in the gut microbiota, particularly the colonic bacteria *Copromorpha*, *RUG420* and *Cryptobacteroides*. These findings underscore the potential role of specific colonic bacteria in regulating IMF deposition and provide a novel approach to enhancing mutton quality through microbial modulation.

## 1. Introduction

Worldwide consumption of livestock meat is steadily increasing. According to FAO statistics, global goat and sheep meat production increased by 57.83% from 78.5 million tons in 2000 to 123.9 million tons in 2023 [1]. With ongoing economic growth and increasing health awareness among consumers, demand for high-quality meat is expected to rise. Intramuscular fat (IMF) content in skeletal muscle tissue is a pivotal determinant of meat quality and palatability, showing a significantly positive association with meat juiciness, shear force, and tenderness. A deeper understanding of the factors regulating IMF development and growth in livestock will facilitate the creation of more effective strategies to optimize intramuscular fat accumulation, thereby enhancing the meat quality and economic value.

Documents have reported that the accumulation of IMF typically occurs at a later stage, often increasing significantly after sexual maturity [2,3,4]. For example, in Japanese Black castrated cattle, the IMF content in the longissimus dorsi increases from 23.7% at 20 months to 38.7% at 25 months and 41.1% at 30 months [5]. The process of IMF deposition is regulated by multiple genes related to lipid metabolism [6,7]. In Kazakh sheep, lipoprotein lipase (*LPL*) expression is strongly and negatively associated with the IMF content in the longissimus dorsi muscle [8,9]. Similarly, in small-tailed Han sheep, *ADIPOQ* expression is strongly and negatively associated with the IMF content in the longissimus dorsi muscle, whereas *PPARGC1A* shows a positive correlation (*r* = 0.923) [10].

In recent years, emerging evidence has indicated an important role played by the gut microbiota in regulating host lipid metabolism. For example, mice receiving “obese” microbiota transferred from Jinhua pigs showed a notable increase in triglyceride (TG) levels and LPL activity compared to those receiving “lean” microbiota from Landrace pigs, indicating that gut microbiota plays a major role in contributing to adiposity in pigs [9]. Dietary supplementation with *Clostridium butyricum* significantly increased the IMF content in the breast muscle of Peking ducks and positively improved meat quality [10]. Additionally, microbial metabolites, particularly short-chain fatty acids (SCFAs), are involved in host lipid metabolism. Evidence suggests that SCFAs not only stimulate the secretion of appetite-suppressing hormones, thereby reducing energy intake and fat accumulation [11,12,13], but also regulate tissue-specific lipid metabolism via AMPK (Adenosine Monophosphate-activated Protein Kinase) activation. For example, acetic acid can activate the AMPKα signaling pathway, which increases lipid oxidation and decreases lipid synthesis in hepatocytes, thereby reducing liver fat accumulation in dairy cows [14]. Nevertheless, few studies have investigated how IMF deposition and gut microbiota vary across different age groups of sheep, or how these factors are interrelated.

Tan sheep, a local breed indigenous to China, is renowned for its tender, low-cholesterol and nutrient-rich meat, making it a superior choice among various types of mutton [15]. In this study, we focused on yearling and mature Tan sheep as research subjects to assess variations in IMF-related traits and gut microbiota. We then analyzed correlations between these factors to identify potential bacterial candidates as future regulatory targets.

## 2. Materials and Methods

### 2.1. Animals

Ten yearlings (1-year-old, 38.29 ± 4.33 kg) and ten mature Tan sheep (4-year-old, 45.82 ± 4.86 kg) from the same herd, both female, privately owned by a local farmer in Maojing Town, Huan County, Gansu Province, were selected as trial animals. The management and feeding practices were identical for all trial Tan sheep. They co-grazed on the same natural grasslands, following a full-day grazing pattern: leaving in the morning and returning in the evening, with no additional supplementary feeding. The pasture vegetation belongs to the true grassland subzone. The annual average temperature ranges from 6.5 to 10 degrees Celsius, and the annual average precipitation is 359.3 mm, primarily concentrated from July to September, accounting for 63% of the total annual precipitation. The primary forage species include *Medicago sativa*, *Agropyron cristatum*, *Artemisia frigida*, *Sonchus arvensis*, and *Stipa bungeana*.

### 2.2. Sample Collection

All twenty Tan sheep were slaughtered on 23 September 2023. Prior to slaughter, they were herded into a quiet environment free from distracting noises or devices and fasted for 12 h with access to water. They were then weighed and slaughtered swiftly and humanely in accordance with Islamic guidelines. These procedures were approved by the Ethics Committee of Gansu Agricultural University (Ethics approval number: GSAU-Eth-AST-2023-037). Each blood sample (5 mL) was collected and centrifuged to separate the serum, which was then used for subsequent analysis of lipid-related indicators. Muscle samples (approximately 20 g each, collected in triplicate) from the shoulder and rump regions were obtained for IMF quantification. Tissue samples (approximately 2 g each, collected in triplicate) from the liver, pancreas, and duodenum, along with gut contents (rumen, abomasum, and colon; approximately 2 mL each, collected in triplicate) were collected for enzyme level measurements. All samples were immediately frozen in liquid nitrogen and stored at −80 °C for further processing.

### 2.3. Determination of IMF Content, Serum Lipid Concentrations, Enzyme Levels, and SCFA Content

IMF content was determined by extracting total fat from muscle using the Soxhlet fat extraction method (ANKOM XT15 Extractor, ANKOM Technology, Macedon, NY, USA). The levels of serum free fatty acids (FFA), TG, total cholesterol (TC), high-density lipoprotein (HDL), low-density lipoprotein (LDL) and very-low-density lipoprotein (VLDL) were measured using ELISA Kits (Shanghai Enzyme-linked Biotechnology Co., Ltd., Shanghai, China). Levels of fatty acid synthase (FAS) and acetyl-CoA carboxylase (ACC) in the digestive glands and gut content, as well as hormone-sensitive lipase (HSL) and LPL, were measured using dedicated kits (Sheep FAS Elisa kit and Sheep ACC Elisa kit, Nanjing Jiancheng Bioengineering Institute, Nanjing, China; and Sheep HSL Elisa kit and Sheep LPL Elisa kit, Fankew, Shanghai Kexing Trading Co., Ltd., Shanghai, China). The concentrations of SCFAs in gut contents were analyzed by gas chromatography using 2-ethylbutyric acid (2-EB) as an internal standard, following the method of Tangerman A [16], with the Agilent 8890 N Network Gas Chromatograph (Agilent Technologies, Santa Clara, CA, USA).

### 2.4. 16S rRNA Analysis

#### 2.4.1. Microbial DNA Extraction and PCR Amplification

Microbial DNA was extracted from gut contents using the E.Z.N.A. ^®^ Stool DNA Kit (Omega Bio-tek, Norcross, GA, USA) following the manufacturer’s instructions. The complete bacterial 16S rRNA gene was amplified using PCR primers 27F (5′-AGRGTTYGATYMTGGCTCAG-3′) and 1492R (5′-RGYTACCTTGTTACGACTT-3′), with each sample containing a unique eight-base barcode.

#### 2.4.2. Sequencing and Data Processing

Amplified DNA was used to construct SMRTbell libraries following the manufacturer’s protocol (Pacific Biosciences, Menlo Park, CA, USA). The libraries were then sequenced on a dedicated PacBio Sequel II system using the sequencing Kit 2.0 chemistry. Raw reads were processed with SMRT link analysis software (v.9.0) to yield demultiplexed circular consensus sequences [17]. Sequences outside the length range of 800 to 2500 base pairs, as well as those failing quality checks and containing > 10 consecutive identical bases, were filtered out. Barcodes, primer sequences, and chimeric sequences were also removed. High-quality sequences were clustered into operational taxonomic units (OTUs) using UPARSE (v.7.1) at a 98.65% similarity threshold, with residual chimeric sequences removed by UCHIME [18,19]. Taxonomic assignment was conducted against the Greengenes2 16S rRNA database using the UCLUST algorithm (v.1.2.22q) with a confidence level at 80% [20,21].

#### 2.4.3. Alpha and Beta Diversity Analyses

Alpha diversity was estimated using Chao1 and Shannon indices, with higher values indicating greater diversity. Beta diversity was assessed through principal coordinate analysis (PCoA) and non-metric multidimensional scaling (NMDS) based on the Bray–Curtis dissimilarity metric. In the resulting ordination plots, samples that cluster closely together exhibit similar microbial community structures, whereas greater separation indicates more distinct profiles.

#### 2.4.4. Functional Prediction of Microbiota

Functional profiles of the microbiota in each group were predicted using the Phylogenetic Investigation of Communities (PICRUSt2) program with reference to the Kyoto Encyclopedia of Genes and Genomes (KEGG) database [22]. OTU abundance data were converted into BIOM files using the make.biom script available in Mothur and aligned with Greengenes OTU IDs, to serve as inputs for PICRUSt2 [23].

### 2.5. Statistical Analysis

Differences in IMF content, lipid levels, lipid-metabolizing enzyme contents and SCFA contents between Tan sheep of different ages were measured using an independent sample *t*-test in SPSS (v.27.0), with *p* < 0.05 considered statistically significant. Chao1 and Shannon diversity indices were analyzed using Mothur (v.1.21.1) [23]. One-way permutational analysis of variance (PERMANOVA) was employed to evaluate statistical significance between the two age groups. Principal coordinate analysis (PCoA) based on the Bray–Curtis dissimilarity matrix was performed using the Phyloseq package [24,25]. Non-metric multidimensional scaling (NMDS) based on the weighted UniFrac distance matrix was performed using the vegan package [26,27]. Differences in microbial genera were evaluated using the *t*-test, while the Wilcoxon rank–sum test was used to assess differences in functional pathways. Associations between age, IMF deposition, microbiota, lipid levels, and SCFA content were analyzed using Spearman correlation analyses. Significant connections were visualized as networks using Cytoscape (v.3.10.1). The absolute value of the correlation coefficients is greater than 0.45 with an FDR < 0.05, indicating a moderate and statistically significant relationship. Meanwhile, |*r*| > 0.6 with an *FDR* < 0.05 indicates a strong and significant correlation between factors.

## 3. Results

### 3.1. Variations in IMF Content Between Yearling and Mature Tan Sheep

**Figure 1:**
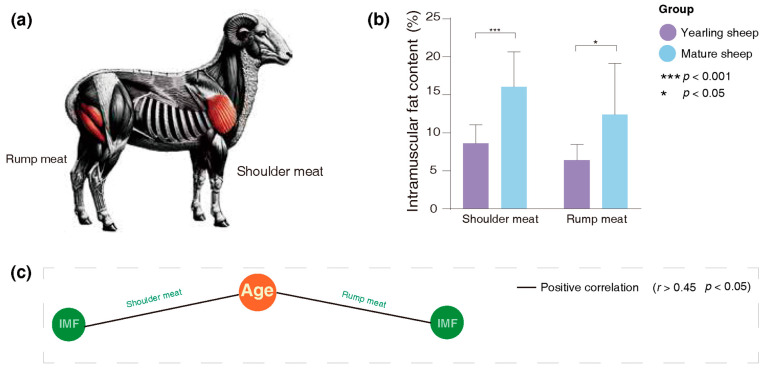
Variation in IMF content between yearling and mature Tan sheep. (**a**) Location of the muscles sampled. (**b**) Differences in IMF content of shoulder meat and rump muscles between yearling and mature sheep. (**c**) Correlation network analysis between age and IMF content in shoulder meat and rump muscles.

IMF content was assessed in the shoulder and rump muscles, with their locations shown in Figure 1a. IMF levels differed significantly between yearling and mature sheep. Mature sheep had higher IMF in shoulder (16.1% vs. 8.6%, *p* < 0.001) and rump (12.2% vs. 6.4%, *p* = 0.024) muscles compared with yearlings (Figure 1b and Supplementary Table S1). Age was strongly and positively correlated with IMF content (*r* > 0.6, *FDR* < 0.05, Figure 1c and Supplementary Table S1).

### 3.2. Differences in Lipid Levels and Organ Enzyme Contents of Yearling and Mature Tan Sheep

**Figure 2:**
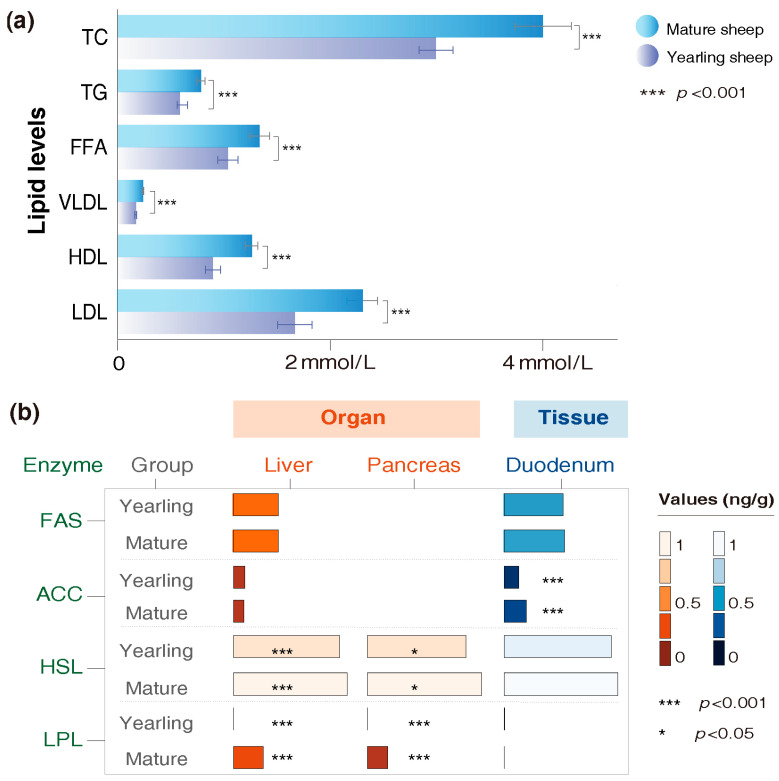
Differences in lipid levels and enzyme contents between yearling and mature Tan sheep. (**a**) Comparison of serum lipid levels in yearling and mature sheep. (**b**) Comparison of enzyme contents of yearling and mature sheep. Y represents yearling Tan sheep. M represents mature Tan sheep.

Serum lipid levels and enzyme contents differed between yearling and mature sheep. Mature sheep had higher serum levels of TC, TG, FFA, LDL, HDL and VLDL compared with yearlings (*p* < 0.001, Figure 2a and Supplementary Table S2). Among the enzymes, the concentrations of HSL and LPL in the liver and pancreas (*p* < 0.05), as well as ACC in the duodenum (*p* < 0.01), were significantly increased in mature sheep relative to yearlings (Figure 2b and Supplementary Table S3). FAS concentrations did not differ significantly between the two age groups.

### 3.3. Variations in SCFA and Enzyme Concentrations in Gut Contents Between Yearling and Mature Tan Sheep

**Figure 3:**
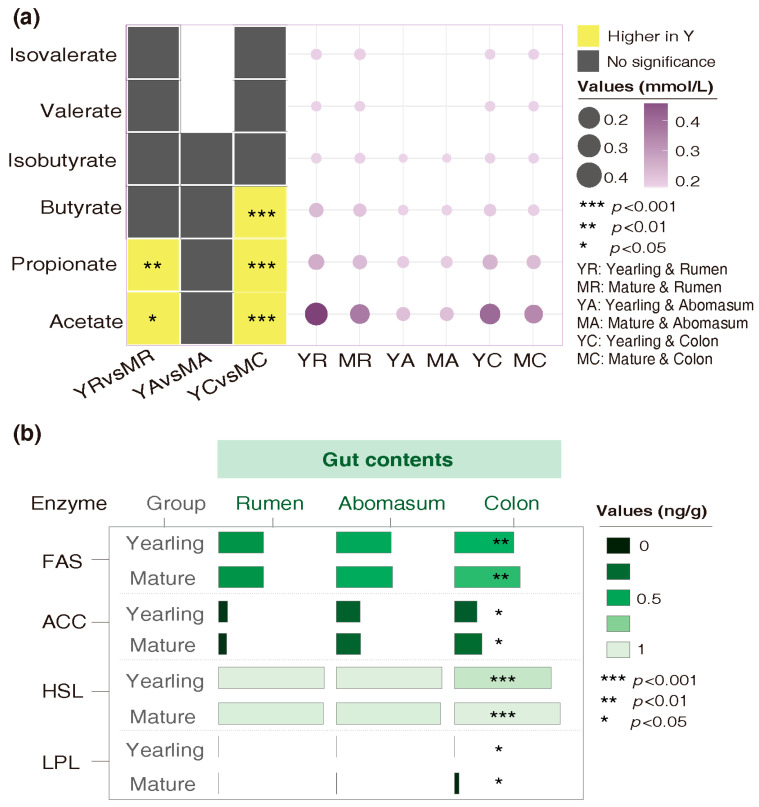
Variations in SCFAs and lipid-metabolizing enzyme concentrations of gut contents of the yearling and mature Tan sheep. (**a**) Comparison of SCFA concentrations in yearling and mature sheep. (**b**) Comparison of levels of key lipid-metabolizing enzymes in yearling and mature sheep.

The concentrations of SCFAs and enzymes in the rumen, abomasum, and colon differed between yearling and mature sheep. In mature sheep, acetate (*p* = 0.044) and propionate (*p* = 0.004) in the ruminal and colonic contents, and butyrate in the colonic chime (*p* < 0.001), decreased significantly (Figure 3a and Supplementary Table S4). Concentrations of FAS (*p* = 0.003) and ACC (*p* = 0.022), as well as HSL (*p* < 0.001) and LPL (*p* = 0.037) in the colonic chime, were significantly higher in mature sheep compared with yearlings (Figure 3b and Supplementary Table S3).

### 3.4. Variations in Gut Microbial Diversity and Functional Profiles Between Yearling and Mature Tan Sheep

**Figure 4:**
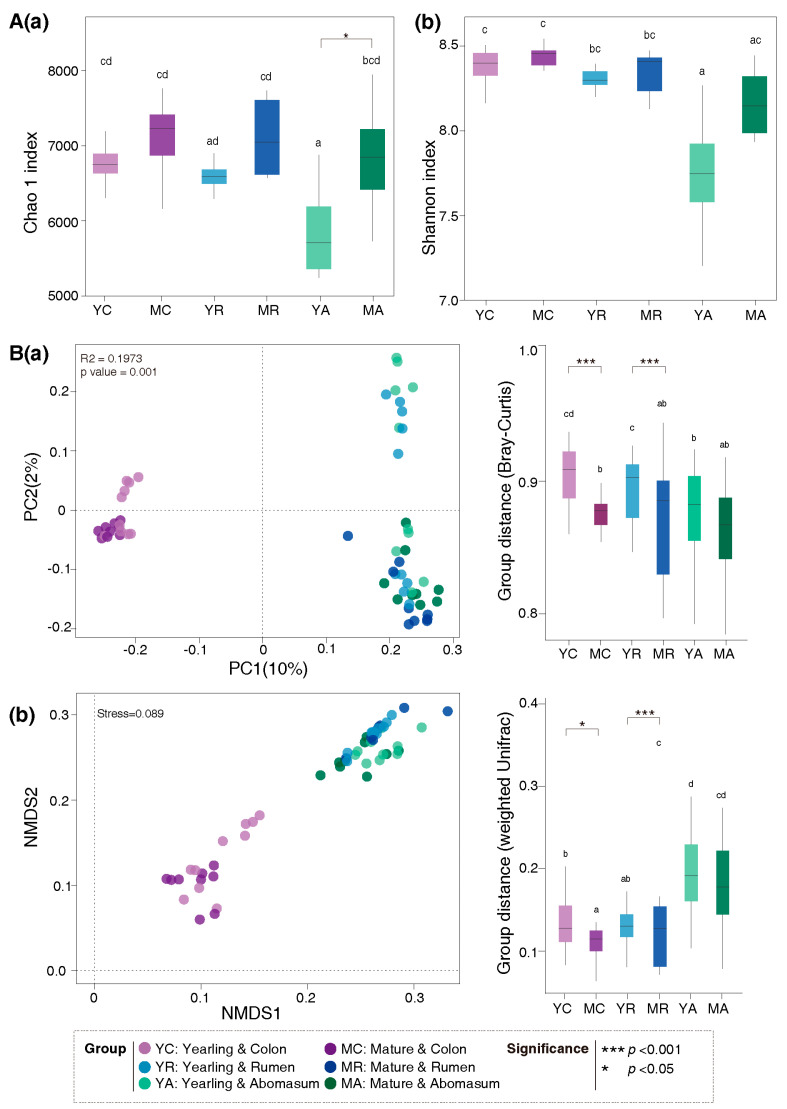
Differences in gut microbiota diversity between yearling and mature Tan sheep. (**A**) Alpha diversity: (**a**) Chao 1 index; (**b**) Simpson index. (**B**) Beta diversity: (**a**) PCoA analysis based on Bray–Curtis distance; (**b**) NMD analysis based on weighted UniFrac distance.

Alpha and beta diversity of gut microbiota were assessed based on the OTU abundance (See methods). The Chao 1 index in the abomasum was significantly higher in mature sheep compared with yearlings (Figure 4A). PCoA and NMDS analyses revealed a clear separation between age groups, particularly in the rumen and colon microbiota (Figure 4B).

**Figure 5:**
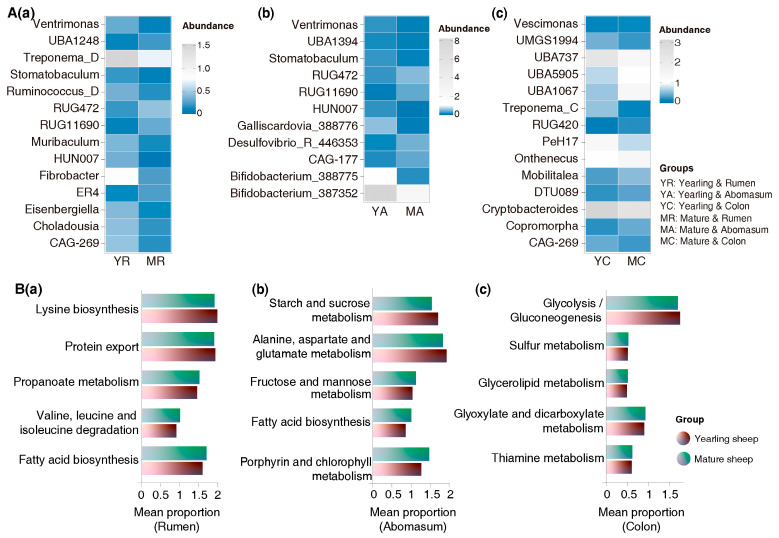
Differences in gut microbiota composition and functional profiles between yearling and mature Tan sheep. (**A**) Microbiota composition: (**a**) ruminal microbiota; (**b**) abomasal microbiota; (**c**) colonic microbiota. (**B**) Functional profiles of specific microbiota: (**a**) functional profiles in rumen; (**b**) functional profiles in abomasum; (**c**) functional profiles in colon.

A total of 14 bacterial taxa in the rumen, 11 in the abomasum, and 14 in the colon differed significantly between yearling and mature sheep. In mature sheep, the abundances of *RUG11690* and *RUG472* in the rumen and abomasum, and *UBA5905*, *Copromorpha*, and *RUG420* in the colon, were significantly higher (Figure 5A). Conversely, the abundances of *Fibrobacter* and *Treponema_D* in the rumen, *Bifidobacterium_387352* and *Bifidobacterium_388775* in the abomasum, and *Cryptobacteroides* and *Treponema_C* in the colon were lower in mature sheep. *Ventrimonas* and *HUN007* in the rumen and abomasum, as well as *CAG-269* in the rumen and colon, were also reduced in mature sheep compared with yearlings.

A functional prediction of microbiota revealed that bacterial taxa in mature sheep had higher lipid metabolism potential. Notably, ruminal microbiota showed increased fatty acid biosynthesis (*p* = 0.0434), while abomasal microbiota exhibited enhanced glycerolipid metabolism (*p* = 0.0101) (Figure 5B).

### 3.5. Correlation of IMF Deposition Indicators with Gut Microbiota

**Figure 6:**
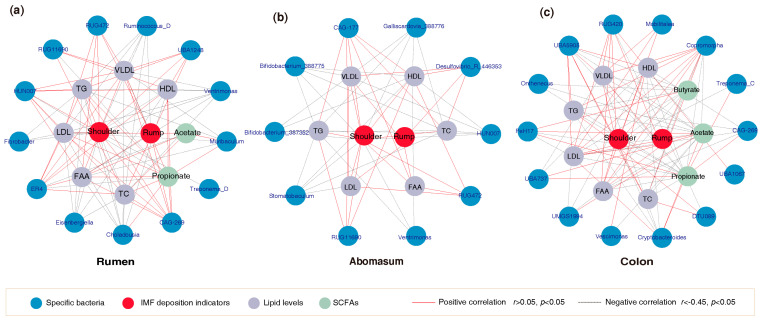
Correlation networks between specific gut bacterial taxa and indicators of IMF content, serum lipid levels, and SCFAs. (**a**) Rumen; (**b**) abomasum; (**c**) colon.

Spearman correlation analysis revealed associations among IMF content in shoulder and rump muscles, serum lipid levels, gut microbiota, and SCFAs. In the rumen, the abundance of *CAG-269* was moderately and negatively correlated with IMF content in both shoulder and rump muscles (*r* < −0.45, *FDR* < 0.05), and TG, TC, FFA, VLDL, HDL and LDL levels (*r* < −0.45, *FDR* < 0.05), but strongly and positively associated with propionate content (*r* = 0.654, *FDR* < 0.05) (Figure 6a and Supplementary Table S5a). In contrast, the abundance of *RUG472* was moderately and positively associated with the IMF content in shoulder and rump muscles (*r* > 0.45, *FDR* < 0.05), and with FFA, VLDL and LDL levels (*r* > 0.45, *FDR* < 0.05) (Figure 6a). In the abomasum, the abundances of *Bifidobacterium_387352* and *Bifidobacterium_388775* were moderately and negatively correlated with IMF content in shoulder muscle, and with TG, TC, VLDL and HDL level (*r* < −0.45, *FDR* < 0.05) (Figure 6b, and Supplementary Table S5b). In the colon, several bacterial taxa were associated with IMF deposition (Figure 6c and Supplementary Table S5c). The abundance of *Cryptobacteroides* was strongly and negatively correlated with the IMF content in the shoulder muscle (*r* < −0.6, *FDR* < 0.05) and moderately negatively correlated with TG and three lipoprotein levels (*r* < −0.45, *FDR* < 0.05), whereas it showed strong and positive correlations with acetate and propionate content (*r* > 0.6, *FDR* < 0.05). *Copromorpha* and *RUG420* abundances were moderately and positively correlated with IMF content in both shoulder and rump muscles (*r* > 0.45, *FDR* < 0.05), and with FFA and three lipoprotein levels (*r* > 0.45, *FDR* < 0.05), but moderately and negatively correlated with propionate (*r* < −0.45, *FDR* < 0.05). The abundance of *UBA5905* was moderately and positively correlated with IMF content in shoulder muscle (*r* > 0.45, *FDR* < 0.05), and with lipid levels excluding FFA and three lipoproteins (*r* > 0.45, *FDR* < 0.05), but moderately and negatively correlated with acetate, propionate and butyrate levels (*r* < −0.45, *FDR* < 0.05).

## 4. Discussion

IMF deposition is influenced by an animal’s age [28,29,30]. Studies have shown that IMF content in the longissimus dorsi muscle of goats varies with growth, peaking at 24 months of age [28]. Similarly, IMF content in the longissimus dorsi muscle of wild red deer increases with slaughter age, ranging from 0.05% in the youngest deer (≤26 months) to 0.34% in the oldest deer (≥42 months) [30]. These results suggest that IMF deposition increases with age, which may be related to the upregulation of key lipid transport proteins and the activation of associated signaling pathways that facilitate IMF accumulation. This research further confirms that mature Tan sheep exhibit a higher IMF content compared to yearlings. Interestingly, the muscle tissues analyzed in this study are the shoulder and rump muscles, rather than the longissimus dorsi muscle, which is more commonly examined. This approach broadens our understanding of fat deposition characteristics in muscles from different anatomical locations during animal growth.

The increase in IMF deposition with age may be related to changes in physiological metabolic activity. Evidence suggests that serum levels of triacylglycerol molecular species are higher in elderly individuals compared to young ones [31,32]. Similar results were obtained in this study, which demonstrated elevated lipid levels in mature Tan sheep. This may be attributed to a further decline in metabolic capacity and a slower rate of lipid clearance with aging. Increased concentrations of TG and TC in the bloodstream facilitate the uptake of these substances by adipocytes, thereby promoting fat deposition [33,34,35]. Our observation that mature sheep exhibit higher IMF content and lipid levels is consistent with the above findings, indicating that age-dependent changes in lipid levels are physiologically important for promoting intramuscular fat deposition.

Enzymes are key regulators of physiological metabolic processes involved in lipid metabolism [36]. A previous study demonstrated that increased gene expression of ACC and FAS promotes the synthesis and accumulation of IMF, thereby enhancing IMF content in beef cattle [37]. ACC and FAS are rate-limiting enzymes in fatty acid synthesis [38]. Additionally, HSL catalyzes the TG into glycerol and FFA [39]. LPL catalyzes the breakdown of TG and phospholipids into FFA [40]. In this study, mature Tan sheep exhibited a significantly higher abundance of LPL and HSL in the liver and pancreas compared to yearling sheep. These findings indicate that the increased enzyme levels correspond with the higher IMF content in mature sheep, underscoring the coordinated role of these enzymes in promoting fat deposition and supporting superior meat production. Additionally, this study examined the levels of FAS, ACC, HSL, and LPL in gut content to identify biomarkers related to host metabolism, despite the fact that measuring these intracellular enzymes is not a conventional practice. Significant changes in enzyme levels were observed in colonic contents, suggesting their potential value as biomarkers for modulating IMF deposition.

The composition and structure of gut microbiota are influenced by the host’s age [41,42]. In this study, differences in gut microbial diversity between yearling and mature sheep were observed, particularly in the rumen and colon, indicating that specific ruminal and colonic bacteria may be involved in the progression of later fat deposition. Functional analyses further revealed that specific bacterial taxa were closely correlated with lipid metabolism pathways, suggesting that age-related microbial shifts may contribute to variations in IMF deposition. Moreover, the concentrations of acetate and propionate in ruminal and colonic contents changed with sheep growth. Mature sheep exhibited lower levels of these SCFAs compared to yearling sheep. Previous studies have documented that SCFAs act as agonists for the AMPK and PPAR (Peroxisome Proliferator-activated Receptor Gamma Coactivator-1 αlpha) pathways in muscle tissue, stimulating PGC-1α (Peroxisome Proliferator-activated Receptor Gamma Coactivator-1 αlpha) expression, promoting FFA oxidation, and suppressing fat accumulation [43,44,45]. These findings are consistent with our observation that mature sheep had a significant reduction in SCFA content, accompanied by an increase in IMF content.

Spearman correlation analysis further suggested that several specific bacterial taxa may be associated with IMF content, SCFAs, and lipid levels. For example, the abundance of *Bifidobacterium_387352* and *Bifidobacterium_388775* in the abomasum were negatively correlated with IMF content in shoulder muscle, and with TG, TC, VLDL and HDL levels. Both *Bifidobacterium_387352* and *Bifidobacterium_388775* belong to the Bifidobacteriaceae family, which has been reported to lower serum TC and TG levels, upregulate thermogenic genes and lipolytic gene expression, and suppress lipogenic gene activation, thereby affecting lipid deposition [46,47,48,49]. Similarly, the abundance of *Cryptobacteroides* was negatively correlated with IMF content in shoulder muscle, and with the levels of TG and three lipoproteins, but positively correlated with acetate and propionate concentrations. Members of the *Cryptobacteroides* genus possess numerous carbohydrate-active enzymes that promote SCFA production [50]. These metabolites not only stimulate the secretion of appetite-suppressing hormones, thereby reducing energy intake and fat accumulation [14,15,16], but also regulate tissue-specific lipid metabolism via AMPK activation. For example, acetic acid can activate the AMPKα signaling pathway to decrease lipid synthesis in hepatocytes, thereby reducing liver fat accumulation. Propionate downregulates hepatic lipogenic genes to lower TG synthesis [51].

Our findings that age-related shifts in gut microbiota are associated with IMF deposition in Tan sheep offer potential strategies for regulating IMF deposition. The identified bacterial taxa warrant further investigation, and future studies should aim to isolate them and verify their functions in IMF regulation, ideally using germ-free animal models under controlled experimental conditions.

## 5. Conclusions

This study demonstrated that mature Tan sheep exhibit significantly higher IMF content in shoulder and rump muscles, accompanied by elevated serum lipid levels, compared with yearling sheep. In contrast, concentrations of acetic acid and propionate in the rumen and colon decreased in mature sheep. Gut bacterial communities differed between age groups. The specific bacterial taxa in the colon, including *Copromorpha*, *RUG420* and *Cryptobacteroides*, were associated with IMF deposition, likely through their influence on acetate and propionate production. These findings suggest that age-related shifts in gut microbiota contribute to variations in IMF deposition. However, the cross-sectional design limits causal inference regarding whether microbial changes drive or result from host aging. Additionally, individual variability, sample size, and reliance on PICRUSt2 require further validation. In future research, we will isolate specific candidate bacteria to verify their effects on intramuscular fat deposition.

## Data Availability

The raw sequence data of 16S rRNA have been submitted to the NCBI database under accession number of PRJNA1219213, the following is the link to the website: http://www.ncbi.nlm.nih.gov/bioproject/1219213, accessed on 10 February 2026. They are openly available in the public repository.

## Supplementary Materials

The following supporting information can be downloaded at: https://www.mdpi.com/article/10.3390/ani16040661/s1, Table S1: IMF contents in shoulder and rump muscles, Spearman correlation between IMF contents and age; Table S2: Serum lipid levels (mmol/L); Table S3: Abundance of lipid-metabolizing enzymes (ng/g); Table S4: Concentrations of SCFAs in gut contents (ug/g); Table S5a: Correlations between specific gut bacterial taxa and IMF deposition indicators (In rumen); Table S5b: Correlations between specific gut bacterial taxa and IMF deposition indicators (In abomasum); Table S5c: Correlations between specific gut bacterial taxa and IMF deposition indicators (In colon).

## Abbreviations

The following abbreviations are used in this manuscript:

2-EB	2-Ethylbutyric acid
AMPK	Adenosine Monophosphate-activated Protein Kinase
ACC	Acetyl-CoA Carboxylase
FAS	Fatty Acid Synthase
FFA	Free Fatty Acids
HDL	High-density lipoprotein
HSL	Hormone-Sensitive Lipase
IMF	Intramuscular Fat
KEGG	Kyoto Encyclopedia of Genes and Genomes
LDL	Low-density lipoprotein
LPL	Lipoprotein Lipase
NMDS	Non-metric Multidimensional Scaling
OTUs	Operational Taxonomic Units
PCoA	Principal Coordinate Analysis
PGC-1α	Peroxisome Proliferator-activated Receptor Gamma Coactivator-1 αlpha
PICRUSt2	Phylogenetic Investigation of Communities by Reconstruction of Unobserved States 2
PPAR	Peroxisome Proliferator-Activated Receptor
SCFAs	Short-Chain Fatty Acids
TC	Triglycerides
TG	Total Cholesterol
VLDL	Very-low-density lipoprotein
