# Supplementary material for: Gut Microbiome Signatures of Aging Associated with Intramuscular Fat Deposition in Tan Sheep
Source: Animals (Basel). 2026 Feb 19;16(4):661. doi: 10.3390/ani16040661 (PMC12937419; doi:10.3390/ani16040661)
Supplement: Supplementary file 1 [file animals-16-00661-s001.zip › Supplementary Table S1.pdf]

Supplementary Table S1

IMF contents in shoulder and rump muscles

Age groups	IMF content (%)	
	Shoulder muscle	Rump muscle
1		5.02
1	11.80	6.45
1	6.96	6.84
1	12.14	7.31
1	7.09	4.72
1	8.24	11.30
1	8.97	6.23
1	8.73	6.69
1	9.24	5.99
1	4.16	3.21
4	16.42	7.57
4	14.07	13.25
4	20.63	6.14
4	25.99	12.07
4	16.33	8.79
4	12.14	19.70
4	9.85	7.51
4	15.58	6.03
4	12.78	16.48
4	16.83	26.45

Spearman correlation between IMF contents and age

Var1	Var2	Correlation	P value	FDR value
Age	Shoulder muscle	0.818	<0.001	<0.001
Age	Rump muscle	0.607	0.005	0.005
