# Supplementary material for: Gut Microbiome Signatures of Aging Associated with Intramuscular Fat Deposition in Tan Sheep
Source: Animals (Basel). 2026 Feb 19;16(4):661. doi: 10.3390/ani16040661 (PMC12937419; doi:10.3390/ani16040661)
Supplement: Supplementary file 1 [file animals-16-00661-s001.zip › Supplementary Table S2.pdf]

**Supplementary Table S2**

Serum lipid levels (mmol/L)

| Age | TG | TC | FFA | VLDL | HDL | LDL |
|---|---|---|---|---|---|---|
| 1 | 0.5341 | 2.9474 | 0.9419 | 0.1788 | 0.9963 | 1.4532 |
| 1 | 0.5475 | 3.0163 | 1.0651 | 0.1676 | 0.767 | 1.8891 |
| 1 | 0.5273 | 3.0232 | 1.0981 | 0.169 | 0.8935 | 1.8149 |
| 1 | 0.6292 | 2.7511 | 0.9059 | 0.1774 | 0.9864 | 1.5823 |
| 1 | 0.6607 | 3.2099 | 1.018 | 0.1662 | 0.937 | 1.6243 |
| 1 | 0.6095 | 3.2917 | 1.026 | 0.1788 | 0.8164 | 1.4887 |
| 1 | 0.5553 | 2.9302 | 1.0581 | 0.1634 | 0.9192 | 1.6437 |
| 1 | 0.518 | 3.0077 | 1.1912 | 0.176 | 0.8619 | 1.8633 |
| 1 | 0.6297 | 2.876 | 0.9229 | 0.1718 | 0.9014 | 1.521 |
| 1 | 0.5713 | 2.8863 | 1.1562 | 0.1746 | 0.8915 | 1.7922 |
| 4 | 0.7347 | 3.9356 | 1.3193 | 0.2402 | 1.3047 | 2.267 |
| 4 | 0.8132 | 3.7824 | 1.3584 | 0.2333 | 1.2612 | 2.4187 |
| 4 | 0.8225 | 4.1775 | 1.4444 | 0.2374 | 1.3501 | 2.5221 |
| 4 | 0.8334 | 4.0372 | 1.3123 | 0.2584 | 1.2809 | 2.3767 |
| 4 | 0.7672 | 3.9494 | 1.3754 | 0.243 | 1.2631 | 2.2928 |
| 4 | 0.7626 | 3.958 | 1.3674 | 0.243 | 1.186 | 2.3509 |
| 4 | 0.7512 | 3.9804 | 1.4294 | 0.2402 | 1.2849 | 2.0215 |
| 4 | 0.8174 | 4.5778 | 1.1331 | 0.2486 | 1.2612 | 2.2799 |
| 4 | 0.7569 | 3.6188 | 1.2793 | 0.2458 | 1.1663 | 2.1701 |
