# Supplementary material for: Gut Microbiome Signatures of Aging Associated with Intramuscular Fat Deposition in Tan Sheep
Source: Animals (Basel). 2026 Feb 19;16(4):661. doi: 10.3390/ani16040661 (PMC12937419; doi:10.3390/ani16040661)
Supplement: Supplementary file 1 [file animals-16-00661-s001.zip › Supplementary Table S3.pdf]

Supplementary Table S3

Abundance of lipid-metabolizing enzymes (ng/g)

Type	Age	FAS	ACC	HSL	LPL
Liver	1	103.7721	7.2483	2123.2573	3.9049
Liver	1	103.6302	5.9723	2364.8003	4.0301
Liver	1	103.3925	9.0869	1999.6195	3.6147
Liver	1	104.0625	7.9265	2397.2815	3.9847
Liver	1	104.5745	9.6310	1899.7031	4.3892
Liver	1	101.2127	6.6622	1855.2243	3.9626
Liver	1	101.7925	9.0587	2023.4569	
Liver	1	102.5172	7.4078	1982.5642	
Liver	1	101.8480			
Liver	4	101.2745	6.7532	3046.4473	20.4271
Liver	4	102.9904	7.3205	3141.1360	22.5346
Liver	4	101.7226	5.8934	3224.3144	22.9025
Liver	4	101.2036	8.6129	3863.0190	23.8684
Liver	4	101.6782	8.7977	3568.7245	27.8119
Liver	4	102.3390	6.9753	3380.8604	25.5923
Liver	4	103.4864	8.2190	2908.0634	23.3380
Liver	4	102.8347	7.2360	3043.6186	22.1104
Liver	4	102.8030		2888.6469	20.6913
Pancreas	1	NA	NA	2138.4521	14.4229
Pancreas	1	NA	NA	1988.4629	16.6565
Pancreas	1	NA	NA	1448.4489	16.4896
Pancreas	1	NA	NA	2130.2400	11.4898
Pancreas	1	NA	NA	1374.7498	11.7607
Pancreas	1	NA	NA	1903.3909	7.6453
Pancreas	1	NA	NA	1157.2366	
Pancreas	4	NA	NA	2057.2709	17.5710
Pancreas	4	NA	NA	1673.9274	36.1845
Pancreas	4	NA	NA	3301.4375	42.1704
Pancreas	4	NA	NA	5524.3951	47.8789
Pancreas	4	NA	NA	6356.4060	29.3518
Pancreas	4	NA	NA	2758.9373	37.9244
Pancreas	4	NA	NA	4434.4942	
Duodenum	1	152.1388	14.3463	1050.2430	11.9292
Duodenum	1	123.1956	21.5725	1049.5656	8.0744
Duodenum	1	137.0991	20.3177	884.5082	12.4589

Type	Age	FAS	ACC	HSL	LPL
Duodenum	1	134.4019	13.4332	811.8991	5.6244
Duodenum	1	93.6650	19.5230	1175.3995	8.9219
Duodenum	1	90.2325	13.9382	844.4027	9.8064
Duodenum	1	123.1956	18.0487	882.1476	10.2574
Duodenum	1	111.7897	20.6024	929.1031	11.3934
Duodenum	1			1052.9486	8.1447
Duodenum	4	124.4287	23.2548	715.9712	6.5224
Duodenum	4	137.7816	26.6259	843.5833	5.8075
Duodenum	4	107.6878	24.4915	1176.0159	9.5053
Duodenum	4	127.2469	28.1860	935.8553	8.0567
Duodenum	4	154.4120	25.7224	1497.0963	11.1450
Duodenum	4	143.3614	23.4524	1808.1783	12.6519
Duodenum	4	105.0374	22.2410	1894.3189	12.9537
Duodenum	4			1765.9840	8.8837
Duodenum	4			1055.6483	
Rumen content	1	46.9436	5.6284	1085.9657	5.2103
Rumen content	1	61.4708	10.9794	990.6658	6.3229
Rumen content	1	67.2557	7.0725	969.6459	5.6759
Rumen content	1	50.7166	9.3093	973.0409	7.8798
Rumen content	1	61.8178	11.5759	1047.0094	7.5656
Rumen content	1	56.9674	9.9466	1065.9924	6.8711
Rumen content	1	57.4519	11.9640	1125.4530	6.6997
Rumen content	1	47.8973	15.3669	1031.5554	8.1108
Rumen content	1	55.8520	7.1460	989.6352	7.6546
Rumen content	1	60.7827	13.6067	1029.8008	4.0882
Rumen content	4	42.7801	8.5878	864.8443	8.2094
Rumen content	4	41.4183	10.8988	1133.4804	6.3963
Rumen content	4	50.7166	12.1159	1079.4159	5.9367
Rumen content	4	61.8178	15.0415	1105.9419	4.0580
Rumen content	4	69.9518	8.6452	950.6878	5.8619
Rumen content	4	54.1404	7.4400	933.0690	5.5524
Rumen content	4	54.7575	8.1431	899.5721	7.7914
Rumen content	4	57.6142	8.1431	874.4687	8.5504
Rumen content	4	82.7794		1147.0927	7.8467
Abomasum content	1	91.5778	14.0974	913.5699	9.6231
Abomasum content	1	73.9910	18.6099	1105.9419	7.1658
Abomasum content	1	89.7969	21.8475	751.0121	3.5542
Abomasum content	1	67.2557	21.9590	1054.1855	4.0796

Type	Age	FAS	ACC	HSL	LPL
Abomasum content	1	78.9234	14.3714	841.2928	3.2022
Abomasum content	1	73.3706	18.3539	902.3294	5.9720
Abomasum content	1	85.3743	13.2532	775.1263	5.7592
Abomasum content	1	60.9543	14.6894	848.8139	5.5684
Abomasum content	1	74.6163	16.4850	912.4778	3.9064
Abomasum content	1	65.7613	14.1790	918.2010	4.0105
Abomasum content	4	87.3125	12.4488	798.9646	4.2850
Abomasum content	4	73.9910	12.5783	948.5647	6.2647
Abomasum content	4	100.7386	13.5069	948.0334	4.4417
Abomasum content	4	64.2994	18.5430	773.5785	4.9615
Abomasum content	4	85.6147	26.0652	579.2832	6.0817
Abomasum content	4	73.1649	17.7601	952.2779	8.3839
Abomasum content	4	90.8105	18.8099	933.8751	3.5409
Abomasum content	4	76.7399	20.6495	857.4298	6.1129
Abomasum content	4	68.3986	12.4928	739.8452	4.1612
Colon content	1	111.4240	16.5045	541.2244	5.3639
Colon content	1	99.8954	19.1395	810.6776	3.6696
Colon content	1	116.5208	19.4568	555.6580	4.0062
Colon content	1	157.2307	18.9937	451.1824	5.6401
Colon content	1	103.3115	15.2125	557.6935	7.4913
Colon content	1	84.6588	16.9744	758.5629	5.3637
Colon content	1	80.2637	13.9807	741.2416	6.3414
Colon content	1	71.1398	14.2955	571.0969	6.9260
Colon content	1	92.6112	13.8100	666.8673	5.1604
Colon content	1		19.1861	662.7943	6.6170
Colon content	4	130.2670	19.7560	1185.8428	6.5792
Colon content	4	113.3094	16.1425	746.7510	5.7335
Colon content	4	124.2445	19.9040	893.8955	5.4040
Colon content	4	145.5493	28.5734	1013.8158	6.0132
Colon content	4	112.3628	18.4080	1084.3110	8.7086
Colon content	4	167.0114	26.6369	939.5882	7.6070
Colon content	4	154.2316	28.1377		7.6025
Colon content	4	145.9524	15.3854		10.2091
Colon content	4	150.8683	21.6335		6.1636
