# Supplementary material for: Gut Microbiome Signatures of Aging Associated with Intramuscular Fat Deposition in Tan Sheep
Source: Animals (Basel). 2026 Feb 19;16(4):661. doi: 10.3390/ani16040661 (PMC12937419; doi:10.3390/ani16040661)
Supplement: Supplementary file 1 [file animals-16-00661-s001.zip › Supplementary Table S4.pdf]

Supplementary Table S4

Concentrations of SCFAs in gut contents（ug/g）

Type	Group	Acetate	Propionate	Butyrate	Isobutyrate	Valerate	Isovalerate
Rumen	1	4305.3921	983.7305	743.8158	114.5537	87.0292	213.7155
Rumen	1	4722.7055	1220.5518	790.4128	93.0891	83.8269	140.7181
Rumen	1	3980.7144	1347.4891	1070.0607	65.3857	96.0460	95.5905
Rumen	1	2433.1977	889.4203	580.6379	78.0633	58.8305	134.3595
Rumen	1	3746.5818	964.1144	617.4141	44.8799	53.8997	64.9480
Rumen	1	3038.8894	1002.5063	619.2697	55.7105	51.7129	78.0001
Rumen	1	2031.1544	670.9221	463.0639	56.4104	53.1594	108.4566
Rumen	4	1891.2200	509.9960	337.1423	93.6505	74.9307	173.5765
Rumen	4	2614.9247	710.2622	427.2498	103.8724	83.6715	203.7512
Rumen	4	3434.2850	834.2323	527.7454	111.1756	86.2471	198.2300
Rumen	4	1910.1094	456.2671	265.3212	76.7373	43.1678	141.5473
Rumen	4	1584.5243	353.2978	217.2489	64.5743	69.9076	125.9190
Rumen	4	2760.9033	724.2705	468.5963	55.3283	49.6934	86.5378
Abomasum	1	677.5685	247.5071	96.8080	9.9238	NA	NA
Abomasum	1	685.7548	206.4145	60.3798	9.3472	NA	NA
Abomasum	1	511.6701	204.3562	57.0621	25.3693	NA	NA
Abomasum	1	494.5913	189.7519	53.2168	44.1800	NA	NA
Abomasum	1	512.4033	203.9635	62.5370	18.2995	NA	NA
Abomasum	1	417.9493	189.6619	63.4947	7.7787	NA	NA
Abomasum	1	578.7133	222.1253	83.3282	39.5426	NA	NA
Abomasum	1	465.3502	189.4704	79.9630	13.9986	NA	NA
Abomasum	4	708.8742	242.3920	71.6620	21.8259	NA	NA
Abomasum	4	392.0465	180.0645	67.2405	7.4066	NA	NA
Abomasum	4	453.7895	155.8066	62.9436	13.4733	NA	NA
Abomasum	4	764.4938	248.0180	107.6090	20.5582	NA	NA
Abomasum	4	378.9617	167.2550	52.2372	20.5548	NA	NA
Abomasum	4	582.5705	215.5969	67.2437	9.4124	NA	NA
Colon	1	2711.6421	738.8780	254.0343	62.0722	64.9258	65.6297
Colon	1	2898.7611	772.4463	200.1916	65.7827	64.5464	79.0769
Colon	1	2521.6976	800.6290	204.2355	95.0688	77.9936	110.9836
Colon	1	2480.8322	711.2104	367.9941	51.4027	61.0514	67.9801
Colon	1	3332.2086	927.0617	253.7129	71.2815	74.8887	73.9161
Colon	1	2191.9418	696.2707	138.5893	64.4966	41.1177	60.7344
Colon	1	2652.7088	882.8041	243.9946	45.5301	50.1163	43.1557
Colon	1	3245.7662	1096.7653	318.2269	75.0673	71.7962	69.1340

Type	Group	Acetate	Propionate	Butyrate	Isobutyrate	Valerate	Isovalerate
Colon	1	2688.7076	923.4813	263.3530	70.7611	75.4335	65.9005
Colon	4	1899.9648	625.1949	172.8372	72.3235	62.7940	84.5779
Colon	4	1917.8291	567.4483	149.5402	56.5277	67.0376	67.4020
Colon	4	1881.1763	587.4947	145.3876	64.7342	57.2443	83.9296
Colon	4	2266.7804	608.7142	158.9731	75.6631	55.0863	77.7602
Colon	4	1172.4228	420.5368	95.0837	55.9912	43.3290	75.1361
Colon	4	1804.1290	573.7408	112.9326	88.6742	44.5337	97.9833
Colon	4	1355.1806	456.7317	89.3903	56.7936	34.6984	60.3545
