# Supplementary material for: Gut Microbiome Signatures of Aging Associated with Intramuscular Fat Deposition in Tan Sheep
Source: Animals (Basel). 2026 Feb 19;16(4):661. doi: 10.3390/ani16040661 (PMC12937419; doi:10.3390/ani16040661)
Supplement: Supplementary file 1 [file animals-16-00661-s001.zip › Supplementary Table S5a.pdf]

Supplementary Table S5a

Correlations between specific gut bacterial taxa and IMF deposition indicators (In rumen)

Var1	Var2	Spearman_rho	P_value	FDR
Shoulder IMF	Rump IMF	0.4772	0.0388	0.0698
Shoulder IMF	LDL（mmol/L）	0.7647	0.0002	0.0030
Rump IMF	LDL（mmol/L）	0.4947	0.0313	0.0609
Shoulder IMF	HDL（mmol/L）	0.7888	0.0001	0.0021
Rump IMF	HDL（mmol/L）	0.3124	0.1928	0.2371
LDL（mmol/L）	HDL（mmol/L）	0.6845	0.0012	0.0084
Shoulder IMF	VLDL（mmol/L）	0.7355	0.0005	0.0050
Rump IMF	VLDL（mmol/L）	0.5815	0.0090	0.0255
LDL（mmol/L）	VLDL（mmol/L）	0.6465	0.0028	0.0126
HDL（mmol/L）	VLDL（mmol/L）	0.7179	0.0005	0.0050
Shoulder IMF	FFA（mmol/L）	0.5459	0.0191	0.0431
Rump IMF	FFA（mmol/L）	0.4842	0.0357	0.0652
LDL（mmol/L）	FFA（mmol/L）	0.8684	0.0000	0.0004
HDL（mmol/L）	FFA（mmol/L）	0.6757	0.0015	0.0093
VLDL（mmol/L）	FFA（mmol/L）	0.6105	0.0055	0.0194
Shoulder IMF	TG（mmol/L）	0.8019	0.0001	0.0016
Rump IMF	TG（mmol/L）	0.3965	0.0928	0.1320
LDL（mmol/L）	TG（mmol/L）	0.7614	0.0002	0.0024
HDL（mmol/L）	TG（mmol/L）	0.7995	0.0000	0.0013
VLDL（mmol/L）	TG（mmol/L）	0.7589	0.0002	0.0024
FFA（mmol/L）	TG（mmol/L）	0.6000	0.0066	0.0213
Shoulder IMF	TC（mmol/L）	0.6594	0.0029	0.0128
Rump IMF	TC（mmol/L）	0.4246	0.0700	0.1077
LDL（mmol/L）	TC（mmol/L）	0.7947	0.0000	0.0014
HDL（mmol/L）	TC（mmol/L）	0.7328	0.0004	0.0038
VLDL（mmol/L）	TC（mmol/L）	0.7668	0.0001	0.0024
FFA（mmol/L）	TC（mmol/L）	0.7596	0.0002	0.0024
TG（mmol/L）	TC（mmol/L）	0.7877	0.0001	0.0016
Shoulder IMF	Acetate（ug/g）	-0.4451	0.1275	0.1685
Rump IMF	Acetate（ug/g）	0.1978	0.5171	0.5621
LDL（mmol/L）	Acetate（ug/g）	-0.5824	0.0367	0.0664
HDL（mmol/L）	Acetate（ug/g）	-0.5495	0.0518	0.0849
VLDL（mmol/L）	Acetate（ug/g）	-0.3824	0.1972	0.2405
FFA（mmol/L）	Acetate（ug/g）	-0.5824	0.0367	0.0664
TG（mmol/L）	Acetate（ug/g）	-0.4451	0.1275	0.1685
TC（mmol/L）	Acetate（ug/g）	-0.5604	0.0463	0.0781
Shoulder IMF	Propionate（ug/g）	-0.5385	0.0576	0.0925
Rump IMF	Propionate（ug/g）	0.0440	0.8866	0.9047

Var1	Var2	Spearman_rho	P_value	FDR
LDL（mmol/L）	Propionate（ug/g）	-0.7637	0.0024	0.0117
HDL（mmol/L）	Propionate（ug/g）	-0.7088	0.0067	0.0213
VLDL（mmol/L）	Propionate（ug/g）	-0.5887	0.0343	0.0635
FFA（mmol/L）	Propionate（ug/g）	-0.7088	0.0067	0.0213
TG（mmol/L）	Propionate（ug/g）	-0.5604	0.0463	0.0781
TC（mmol/L）	Propionate（ug/g）	-0.6538	0.0153	0.0365
Acetate（ug/g）	Propionate（ug/g）	0.9011	0.0000	0.0010
Shoulder IMF	CAG-269	-0.7523	0.0003	0.0035
Rump IMF	CAG-269	-0.4649	0.0449	0.0779
LDL（mmol/L）	CAG-269	-0.7053	0.0007	0.0057
HDL（mmol/L）	CAG-269	-0.6959	0.0009	0.0067
VLDL（mmol/L）	CAG-269	-0.5806	0.0091	0.0257
FFA（mmol/L）	CAG-269	-0.5737	0.0102	0.0279
TG（mmol/L）	CAG-269	-0.6281	0.0040	0.0161
TC（mmol/L）	CAG-269	-0.6456	0.0028	0.0127
Acetate（ug/g）	CAG-269	0.4945	0.0858	0.1244
Propionate（ug/g）	CAG-269	0.6538	0.0153	0.0365
Shoulder IMF	Choladousia	-0.5273	0.0245	0.0525
Rump IMF	Choladousia	-0.1351	0.5814	0.6207
LDL（mmol/L）	Choladousia	-0.6088	0.0057	0.0196
HDL（mmol/L）	Choladousia	-0.6810	0.0013	0.0087
VLDL（mmol/L）	Choladousia	-0.4743	0.0402	0.0718
FFA（mmol/L）	Choladousia	-0.5754	0.0099	0.0276
TG（mmol/L）	Choladousia	-0.5491	0.0149	0.0363
TC（mmol/L）	Choladousia	-0.7158	0.0006	0.0050
Acetate（ug/g）	Choladousia	0.4451	0.1275	0.1685
Propionate（ug/g）	Choladousia	0.6538	0.0153	0.0365
CAG-269	Choladousia	0.5070	0.0267	0.0554
Shoulder IMF	Eisenbergiella	-0.5686	0.0138	0.0345
Rump IMF	Eisenbergiella	-0.1561	0.5233	0.5667
LDL（mmol/L）	Eisenbergiella	-0.6105	0.0055	0.0194
HDL（mmol/L）	Eisenbergiella	-0.6152	0.0051	0.0187
VLDL（mmol/L）	Eisenbergiella	-0.4436	0.0571	0.0922
FFA（mmol/L）	Eisenbergiella	-0.4895	0.0334	0.0623
TG（mmol/L）	Eisenbergiella	-0.5860	0.0084	0.0249
TC（mmol/L）	Eisenbergiella	-0.6772	0.0014	0.0092
Acetate（ug/g）	Eisenbergiella	0.3626	0.2233	0.2701
Propionate（ug/g）	Eisenbergiella	0.5330	0.0607	0.0964
CAG-269	Eisenbergiella	0.6860	0.0012	0.0083
Choladousia	Eisenbergiella	0.8193	0.0000	0.0008
Shoulder IMF	ER4	0.7193	0.0008	0.0057
Rump IMF	ER4	0.1053	0.6680	0.7056

Var1	Var2	Spearman_rho	P_value	FDR
LDL（mmol/L）	ER4	0.5737	0.0102	0.0279
HDL（mmol/L）	ER4	0.7091	0.0007	0.0055
VLDL（mmol/L）	ER4	0.5665	0.0114	0.0301
FFA（mmol/L）	ER4	0.5053	0.0273	0.0558
TG（mmol/L）	ER4	0.4930	0.0320	0.0619
TC（mmol/L）	ER4	0.6035	0.0062	0.0207
Acetate（ug/g）	ER4	-0.6758	0.0112	0.0298
Propionate（ug/g）	ER4	-0.8187	0.0006	0.0053
CAG-269	ER4	-0.6211	0.0045	0.0172
Choladousia	ER4	-0.7614	0.0002	0.0024
Eisenbergiella	ER4	-0.5947	0.0072	0.0228
Shoulder IMF	Fibrobacter	-0.5046	0.0327	0.0619
Rump IMF	Fibrobacter	-0.1930	0.4286	0.4834
LDL（mmol/L）	Fibrobacter	-0.4579	0.0487	0.0807
HDL（mmol/L）	Fibrobacter	-0.3642	0.1253	0.1685
VLDL（mmol/L）	Fibrobacter	-0.2477	0.3066	0.3621
FFA（mmol/L）	Fibrobacter	-0.1439	0.5568	0.5966
TG（mmol/L）	Fibrobacter	-0.5140	0.0244	0.0525
TC（mmol/L）	Fibrobacter	-0.3649	0.1245	0.1682
Acetate（ug/g）	Fibrobacter	0.0440	0.8866	0.9047
Propionate（ug/g）	Fibrobacter	0.2253	0.4593	0.5085
CAG-269	Fibrobacter	0.7158	0.0006	0.0050
Choladousia	Fibrobacter	0.4158	0.0766	0.1138
Eisenbergiella	Fibrobacter	0.6491	0.0026	0.0124
ER4	Fibrobacter	-0.3298	0.1679	0.2107
Shoulder IMF	HUN007	-0.6453	0.0038	0.0158
Rump IMF	HUN007	-0.3660	0.1233	0.1674
LDL（mmol/L）	HUN007	-0.5380	0.0175	0.0410
HDL（mmol/L）	HUN007	-0.7397	0.0003	0.0035
VLDL（mmol/L）	HUN007	-0.7781	0.0001	0.0020
FFA（mmol/L）	HUN007	-0.5029	0.0282	0.0568
TG（mmol/L）	HUN007	-0.6749	0.0015	0.0093
TC（mmol/L）	HUN007	-0.6275	0.0040	0.0161
Acetate（ug/g）	HUN007	0.3356	0.2622	0.3147
Propionate（ug/g）	HUN007	0.6465	0.0170	0.0400
CAG-269	HUN007	0.7442	0.0003	0.0032
Choladousia	HUN007	0.5827	0.0088	0.0255
Eisenbergiella	HUN007	0.5836	0.0087	0.0255
ER4	HUN007	-0.6161	0.0050	0.0187
Fibrobacter	HUN007	0.5248	0.0211	0.0464
Shoulder IMF	Muribaculum	-0.4138	0.0878	0.1260
Rump IMF	Muribaculum	-0.2579	0.2864	0.3396

Var1	Var2	Spearman_rho	P_value	FDR
LDL（mmol/L）	Muribaculum	-0.5018	0.0286	0.0572
HDL（mmol/L）	Muribaculum	-0.4344	0.0631	0.0996
VLDL（mmol/L）	Muribaculum	-0.5112	0.0253	0.0534
FFA（mmol/L）	Muribaculum	-0.5053	0.0273	0.0558
TG（mmol/L）	Muribaculum	-0.4632	0.0458	0.0781
TC（mmol/L）	Muribaculum	-0.3509	0.1408	0.1820
Acetate（ug/g）	Muribaculum	0.6813	0.0103	0.0279
Propionate（ug/g）	Muribaculum	0.6703	0.0122	0.0315
CAG-269	Muribaculum	0.3456	0.1472	0.1888
Choladousia	Muribaculum	0.1789	0.4636	0.5113
Eisenbergiella	Muribaculum	-0.0386	0.8753	0.8993
ER4	Muribaculum	-0.3737	0.1150	0.1576
Fibrobacter	Muribaculum	-0.0123	0.9602	0.9602
HUN007	Muribaculum	0.4555	0.0500	0.0825
Shoulder IMF	RUG11690	0.3581	0.1445	0.1861
Rump IMF	RUG11690	0.0702	0.7753	0.7993
LDL（mmol/L）	RUG11690	0.3140	0.1904	0.2351
HDL（mmol/L）	RUG11690	0.4985	0.0298	0.0593
VLDL（mmol/L）	RUG11690	0.5332	0.0187	0.0429
FFA（mmol/L）	RUG11690	0.4193	0.0739	0.1115
TG（mmol/L）	RUG11690	0.4281	0.0675	0.1049
TC（mmol/L）	RUG11690	0.5526	0.0141	0.0350
Acetate（ug/g）	RUG11690	-0.4780	0.0985	0.1381
Propionate（ug/g）	RUG11690	-0.5714	0.0413	0.0734
CAG-269	RUG11690	-0.0228	0.9262	0.9324
Choladousia	RUG11690	-0.5860	0.0084	0.0249
Eisenbergiella	RUG11690	-0.2140	0.3789	0.4372
ER4	RUG11690	0.6404	0.0031	0.0133
Fibrobacter	RUG11690	0.2211	0.3631	0.4212
HUN007	RUG11690	-0.3572	0.1333	0.1731
Muribaculum	RUG11690	-0.4035	0.0867	0.1250
Shoulder IMF	RUG472	0.5459	0.0191	0.0431
Rump IMF	RUG472	0.6421	0.0030	0.0132
LDL（mmol/L）	RUG472	0.5877	0.0081	0.0247
HDL（mmol/L）	RUG472	0.4046	0.0858	0.1244
VLDL（mmol/L）	RUG472	0.5551	0.0136	0.0345
FFA（mmol/L）	RUG472	0.4579	0.0487	0.0807
TG（mmol/L）	RUG472	0.4158	0.0766	0.1138
TC（mmol/L）	RUG472	0.4211	0.0726	0.1100
Acetate（ug/g）	RUG472	-0.0330	0.9149	0.9241
Propionate（ug/g）	RUG472	-0.3846	0.1944	0.2381
CAG-269	RUG472	-0.6807	0.0013	0.0087

Var1	Var2	Spearman_rho	P_value	FDR
Choladousia	RUG472	-0.4088	0.0823	0.1210
Eisenbergiella	RUG472	-0.5193	0.0227	0.0497
ER4	RUG472	0.3263	0.1727	0.2159
Fibrobacter	RUG472	-0.6667	0.0018	0.0098
HUN007	RUG472	-0.6047	0.0061	0.0206
Muribaculum	RUG472	-0.1123	0.6472	0.6861
RUG11690	RUG472	-0.1140	0.6420	0.6830
Shoulder IMF	Ruminococcus_D	-0.4757	0.0460	0.0781
Rump IMF	Ruminococcus_D	-0.5035	0.0280	0.0567
LDL（mmol/L）	Ruminococcus_D	-0.4579	0.0487	0.0807
HDL（mmol/L）	Ruminococcus_D	-0.3300	0.1677	0.2107
VLDL（mmol/L）	Ruminococcus_D	-0.4910	0.0328	0.0619
FFA（mmol/L）	Ruminococcus_D	-0.4105	0.0808	0.1195
TG（mmol/L）	Ruminococcus_D	-0.4860	0.0349	0.0642
TC（mmol/L）	Ruminococcus_D	-0.4947	0.0313	0.0609
Acetate（ug/g）	Ruminococcus_D	0.2033	0.5053	0.5513
Propionate（ug/g）	Ruminococcus_D	0.2747	0.3637	0.4212
CAG-269	Ruminococcus_D	0.6509	0.0025	0.0121
Choladousia	Ruminococcus_D	0.2088	0.3910	0.4477
Eisenbergiella	Ruminococcus_D	0.4263	0.0687	0.1063
ER4	Ruminococcus_D	-0.1702	0.4861	0.5342
Fibrobacter	Ruminococcus_D	0.5632	0.0121	0.0314
HUN007	Ruminococcus_D	0.4642	0.0452	0.0780
Muribaculum	Ruminococcus_D	0.0333	0.8922	0.9074
RUG11690	Ruminococcus_D	0.1526	0.5328	0.5749
RUG472	Ruminococcus_D	-0.6667	0.0018	0.0098
Shoulder IMF	Stomatobaculum	-0.3975	0.1023	0.1421
Rump IMF	Stomatobaculum	-0.2317	0.3399	0.3983
LDL（mmol/L）	Stomatobaculum	-0.3940	0.0951	0.1345
HDL（mmol/L）	Stomatobaculum	-0.4219	0.0720	0.1097
VLDL（mmol/L）	Stomatobaculum	-0.3748	0.1139	0.1567
FFA（mmol/L）	Stomatobaculum	-0.3308	0.1665	0.2107
TG（mmol/L）	Stomatobaculum	-0.3581	0.1323	0.1725
TC（mmol/L）	Stomatobaculum	-0.5546	0.0137	0.0345
Acetate（ug/g）	Stomatobaculum	0.1868	0.5411	0.5819
Propionate（ug/g）	Stomatobaculum	0.3956	0.1809	0.2242
CAG-269	Stomatobaculum	0.6713	0.0016	0.0098
Choladousia	Stomatobaculum	0.6222	0.0044	0.0171
Eisenbergiella	Stomatobaculum	0.6617	0.0020	0.0103
ER4	Stomatobaculum	-0.3932	0.0959	0.1350
Fibrobacter	Stomatobaculum	0.6687	0.0017	0.0098
HUN007	Stomatobaculum	0.6089	0.0057	0.0196

Var1	Var2	Spearman_rho	P_value	FDR
Muribaculum	Stomatobaculum	0.0921	0.7075	0.7370
RUG11690	Stomatobaculum	0.0140	0.9545	0.9577
RUG472	Stomatobaculum	-0.5906	0.0078	0.0240
Ruminococcus_D	Stomatobaculum	0.5362	0.0180	0.0418
Shoulder IMF	Treponema_D	-0.6388	0.0043	0.0168
Rump IMF	Treponema_D	-0.3632	0.1265	0.1685
LDL（mmol/L）	Treponema_D	-0.6667	0.0018	0.0098
HDL（mmol/L）	Treponema_D	-0.5687	0.0111	0.0296
VLDL（mmol/L）	Treponema_D	-0.5815	0.0090	0.0255
FFA（mmol/L）	Treponema_D	-0.4895	0.0334	0.0623
TG（mmol/L）	Treponema_D	-0.6702	0.0017	0.0098
TC（mmol/L）	Treponema_D	-0.7000	0.0008	0.0062
Acetate（ug/g）	Treponema_D	0.2967	0.3249	0.3823
Propionate（ug/g）	Treponema_D	0.4505	0.1223	0.1668
CAG-269	Treponema_D	0.8175	0.0000	0.0008
Choladousia	Treponema_D	0.5877	0.0081	0.0247
Eisenbergiella	Treponema_D	0.8561	0.0000	0.0004
ER4	Treponema_D	-0.4912	0.0327	0.0619
Fibrobacter	Treponema_D	0.7632	0.0001	0.0024
HUN007	Treponema_D	0.6406	0.0031	0.0133
Muribaculum	Treponema_D	0.1860	0.4459	0.4973
RUG11690	Treponema_D	-0.0719	0.7698	0.7991
RUG472	Treponema_D	-0.6105	0.0055	0.0194
Ruminococcus_D	Treponema_D	0.6105	0.0055	0.0194
Stomatobaculum	Treponema_D	0.7301	0.0004	0.0040
Shoulder IMF	UBA1248	0.5648	0.0146	0.0359
Rump IMF	UBA1248	0.1869	0.4435	0.4973
LDL（mmol/L）	UBA1248	0.4054	0.0850	0.1244
HDL（mmol/L）	UBA1248	0.6067	0.0059	0.0201
VLDL（mmol/L）	UBA1248	0.5580	0.0130	0.0334
FFA（mmol/L）	UBA1248	0.4230	0.0712	0.1089
TG（mmol/L）	UBA1248	0.5248	0.0211	0.0464
TC（mmol/L）	UBA1248	0.5906	0.0078	0.0240
Acetate（ug/g）	UBA1248	-0.3246	0.2792	0.3329
Propionate（ug/g）	UBA1248	-0.4622	0.1118	0.1546
CAG-269	UBA1248	-0.3317	0.1653	0.2107
Choladousia	UBA1248	-0.7442	0.0003	0.0032
Eisenbergiella	UBA1248	-0.5151	0.0240	0.0522
ER4	UBA1248	0.8170	0.0000	0.0008
Fibrobacter	UBA1248	-0.1860	0.4457	0.4973
HUN007	UBA1248	-0.5119	0.0251	0.0533
Muribaculum	UBA1248	-0.1992	0.4136	0.4682

Var1	Var2	Spearman_rho	P_value	FDR
RUG11690	UBA1248	0.8469	0.0000	0.0005
RUG472	UBA1248	0.2080	0.3929	0.4481
Ruminococcus_D	UBA1248	-0.1009	0.6810	0.7138
Stomatobaculum	UBA1248	-0.2230	0.3588	0.4188
Treponema_D	UBA1248	-0.3589	0.1313	0.1720
Shoulder IMF	Ventrimonas	-0.6763	0.0021	0.0103
Rump IMF	Ventrimonas	-0.2106	0.3868	0.4446
LDL（mmol/L）	Ventrimonas	-0.6529	0.0024	0.0118
HDL（mmol/L）	Ventrimonas	-0.8376	0.0000	0.0006
VLDL（mmol/L）	Ventrimonas	-0.4688	0.0429	0.0748
FFA（mmol/L）	Ventrimonas	-0.6003	0.0066	0.0213
TG（mmol/L）	Ventrimonas	-0.7170	0.0006	0.0050
TC（mmol/L）	Ventrimonas	-0.6652	0.0019	0.0099
Acetate（ug/g）	Ventrimonas	0.3686	0.2152	0.2613
Propionate（ug/g）	Ventrimonas	0.5337	0.0603	0.0962
CAG-269	Ventrimonas	0.7082	0.0007	0.0055
Choladousia	Ventrimonas	0.7696	0.0001	0.0023
Eisenbergiella	Ventrimonas	0.7389	0.0003	0.0035
ER4	Ventrimonas	-0.7108	0.0006	0.0054
Fibrobacter	Ventrimonas	0.6301	0.0038	0.0158
HUN007	Ventrimonas	0.6629	0.0020	0.0102
Muribaculum	Ventrimonas	0.1834	0.4523	0.5025
RUG11690	Ventrimonas	-0.3896	0.0991	0.1383
RUG472	Ventrimonas	-0.4713	0.0417	0.0735
Ruminococcus_D	Ventrimonas	0.4309	0.0655	0.1029
Stomatobaculum	Ventrimonas	0.5347	0.0183	0.0423
Treponema_D	Ventrimonas	0.6477	0.0027	0.0125
UBA1248	Ventrimonas	-0.6681	0.0018	0.0098
