# Supplementary material for: Gut Microbiome Signatures of Aging Associated with Intramuscular Fat Deposition in Tan Sheep
Source: Animals (Basel). 2026 Feb 19;16(4):661. doi: 10.3390/ani16040661 (PMC12937419; doi:10.3390/ani16040661)
Supplement: Supplementary file 1 [file animals-16-00661-s001.zip › Supplementary Table S5b.pdf]

Supplementary Table S5b

Correlations between specific gut bacterial taxa and IMF deposition indicators (In abomasum)

Var1	Var2	Spearman_rho	P_value	FDR
Shoulder IMF	Rump IMF	0.4772	0.0388	0.0716
Shoulder IMF	LDL（mmol/L）	0.7647	0.0002	0.0026
Rump IMF	LDL（mmol/L）	0.4947	0.0313	0.0632
Shoulder IMF	HDL（mmol/L）	0.7888	0.0001	0.0019
Rump IMF	HDL（mmol/L）	0.3124	0.1928	0.2290
LDL（mmol/L）	HDL（mmol/L）	0.6845	0.0012	0.0093
Shoulder IMF	VLDL（mmol/L）	0.7355	0.0005	0.0050
Rump IMF	VLDL（mmol/L）	0.5815	0.0090	0.0336
LDL（mmol/L）	VLDL（mmol/L）	0.6465	0.0028	0.0152
HDL（mmol/L）	VLDL（mmol/L）	0.7179	0.0005	0.0051
Shoulder IMF	FFA（mmol/L）	0.5459	0.0191	0.0462
Rump IMF	FFA（mmol/L）	0.4842	0.0357	0.0671
LDL（mmol/L）	FFA（mmol/L）	0.8684	0.0000	0.0001
HDL（mmol/L）	FFA（mmol/L）	0.6757	0.0015	0.0109
VLDL（mmol/L）	FFA（mmol/L）	0.6105	0.0055	0.0243
Shoulder IMF	TG（mmol/L）	0.8019	0.0001	0.0015
Rump IMF	TG（mmol/L）	0.3965	0.0928	0.1297
LDL（mmol/L）	TG（mmol/L）	0.7614	0.0002	0.0022
HDL（mmol/L）	TG（mmol/L）	0.7995	0.0000	0.0015
VLDL（mmol/L）	TG（mmol/L）	0.7589	0.0002	0.0022
FFA（mmol/L）	TG（mmol/L）	0.6000	0.0066	0.0273
Shoulder IMF	TC（mmol/L）	0.6594	0.0029	0.0152
Rump IMF	TC（mmol/L）	0.4246	0.0700	0.1039
LDL（mmol/L）	TC（mmol/L）	0.7947	0.0000	0.0015
HDL（mmol/L）	TC（mmol/L）	0.7328	0.0004	0.0038
VLDL（mmol/L）	TC（mmol/L）	0.7668	0.0001	0.0022
FFA（mmol/L）	TC（mmol/L）	0.7596	0.0002	0.0022
TG（mmol/L）	TC（mmol/L）	0.7877	0.0001	0.0015
Shoulder IMF	Bifidobacterium_387352	-0.5913	0.0097	0.0349
Rump IMF	Bifidobacterium_387352	-0.2421	0.3180	0.3639
LDL（mmol/L）	Bifidobacterium_387352	-0.3754	0.1132	0.1494
HDL（mmol/L）	Bifidobacterium_387352	-0.7100	0.0007	0.0057
VLDL（mmol/L）	Bifidobacterium_387352	-0.5823	0.0089	0.0336
FFA（mmol/L）	Bifidobacterium_387352	-0.3789	0.1096	0.1466
TG（mmol/L）	Bifidobacterium_387352	-0.5211	0.0222	0.0507
TC（mmol/L）	Bifidobacterium_387352	-0.5684	0.0111	0.0370
Shoulder IMF	Bifidobacterium_388775	-0.6471	0.0037	0.0185
Rump IMF	Bifidobacterium_388775	-0.3193	0.1827	0.2197

Var1	Var2	Spearman_rho	P_value	FDR
LDL（mmol/L）	Bifidobacterium_388775	-0.3860	0.1027	0.1413
HDL（mmol/L）	Bifidobacterium_388775	-0.6871	0.0012	0.0091
VLDL（mmol/L）	Bifidobacterium_388775	-0.5718	0.0105	0.0364
FFA（mmol/L）	Bifidobacterium_388775	-0.2737	0.2569	0.2976
TG（mmol/L）	Bifidobacterium_388775	-0.5456	0.0157	0.0426
TC（mmol/L）	Bifidobacterium_388775	-0.4860	0.0349	0.0663
Bifidobacterium_387352	Bifidobacterium_388775	0.8930	0.0000	0.0000
Shoulder IMF	CAG-177	0.5645	0.0147	0.0416
Rump IMF	CAG-177	0.1614	0.5092	0.5466
LDL（mmol/L）	CAG-177	0.5421	0.0165	0.0433
HDL（mmol/L）	CAG-177	0.7477	0.0002	0.0026
VLDL（mmol/L）	CAG-177	0.4295	0.0665	0.1018
FFA（mmol/L）	CAG-177	0.5351	0.0182	0.0460
TG（mmol/L）	CAG-177	0.6088	0.0057	0.0245
TC（mmol/L）	CAG-177	0.5982	0.0068	0.0275
Bifidobacterium_387352	CAG-177	-0.6281	0.0040	0.0190
Bifidobacterium_388775	CAG-177	-0.4930	0.0320	0.0633
Shoulder IMF	Desulfovibrio_R_446353	0.3953	0.1045	0.1418
Rump IMF	Desulfovibrio_R_446353	0.4860	0.0349	0.0663
LDL（mmol/L）	Desulfovibrio_R_446353	0.4491	0.0537	0.0887
HDL（mmol/L）	Desulfovibrio_R_446353	0.5186	0.0229	0.0518
VLDL（mmol/L）	Desulfovibrio_R_446353	0.4892	0.0335	0.0650
FFA（mmol/L）	Desulfovibrio_R_446353	0.4035	0.0867	0.1238
TG（mmol/L）	Desulfovibrio_R_446353	0.4474	0.0548	0.0890
TC（mmol/L）	Desulfovibrio_R_446353	0.5596	0.0127	0.0385
Bifidobacterium_387352	Desulfovibrio_R_446353	-0.6228	0.0044	0.0199
Bifidobacterium_388775	Desulfovibrio_R_446353	-0.6579	0.0022	0.0144
CAG-177	Desulfovibrio_R_446353	0.5825	0.0089	0.0336
Shoulder IMF	Galliscardovia_388776	-0.5728	0.0130	0.0385
Rump IMF	Galliscardovia_388776	-0.3282	0.1701	0.2072
LDL（mmol/L）	Galliscardovia_388776	-0.3730	0.1158	0.1517
HDL（mmol/L）	Galliscardovia_388776	-0.6462	0.0028	0.0152
VLDL（mmol/L）	Galliscardovia_388776	-0.6516	0.0025	0.0152
FFA（mmol/L）	Galliscardovia_388776	-0.2791	0.2473	0.2882
TG（mmol/L）	Galliscardovia_388776	-0.5485	0.0150	0.0420
TC（mmol/L）	Galliscardovia_388776	-0.5599	0.0127	0.0385
Bifidobacterium_387352	Galliscardovia_388776	0.8846	0.0000	0.0000
Bifidobacterium_388775	Galliscardovia_388776	0.9258	0.0000	0.0000
CAG-177	Galliscardovia_388776	-0.5415	0.0167	0.0433
Desulfovibrio_R_446353	Galliscardovia_388776	-0.7556	0.0002	0.0023
Shoulder IMF	HUN007	-0.4915	0.0383	0.0714
Rump IMF	HUN007	-0.2334	0.3361	0.3824

Var1	Var2	Spearman_rho	P_value	FDR
LDL（mmol/L）	HUN007	-0.4125	0.0793	0.1150
HDL（mmol/L）	HUN007	-0.5593	0.0128	0.0385
VLDL（mmol/L）	HUN007	-0.4627	0.0461	0.0803
FFA（mmol/L）	HUN007	-0.2922	0.2247	0.2652
TG（mmol/L）	HUN007	-0.5090	0.0260	0.0562
TC（mmol/L）	HUN007	-0.4581	0.0486	0.0824
Bifidobacterium_387352	HUN007	0.5625	0.0122	0.0385
Bifidobacterium_388775	HUN007	0.6722	0.0016	0.0114
CAG-177	HUN007	-0.5643	0.0118	0.0385
Desulfovibrio_R_446353	HUN007	-0.7135	0.0006	0.0055
Galliscardovia_388776	HUN007	0.6036	0.0062	0.0262
Shoulder IMF	RUG11690	0.5728	0.0130	0.0385
Rump IMF	RUG11690	-0.0246	0.9205	0.9353
LDL（mmol/L）	RUG11690	0.1912	0.4329	0.4727
HDL（mmol/L）	RUG11690	0.5538	0.0139	0.0400
VLDL（mmol/L）	RUG11690	0.5314	0.0192	0.0462
FFA（mmol/L）	RUG11690	0.1825	0.4547	0.4909
TG（mmol/L）	RUG11690	0.4737	0.0405	0.0733
TC（mmol/L）	RUG11690	0.3526	0.1387	0.1745
Bifidobacterium_387352	RUG11690	-0.4351	0.0626	0.0976
Bifidobacterium_388775	RUG11690	-0.3649	0.1245	0.1609
CAG-177	RUG11690	0.2246	0.3553	0.3972
Desulfovibrio_R_446353	RUG11690	0.0140	0.9545	0.9596
Galliscardovia_388776	RUG11690	-0.3572	0.1333	0.1688
HUN007	RUG11690	-0.1027	0.6757	0.7054
Shoulder IMF	RUG472	0.5851	0.0107	0.0364
Rump IMF	RUG472	0.2825	0.2413	0.2830
LDL（mmol/L）	RUG472	0.5070	0.0267	0.0566
HDL（mmol/L）	RUG472	0.5327	0.0189	0.0462
VLDL（mmol/L）	RUG472	0.3970	0.0924	0.1297
FFA（mmol/L）	RUG472	0.4754	0.0397	0.0724
TG（mmol/L）	RUG472	0.4070	0.0837	0.1205
TC（mmol/L）	RUG472	0.4404	0.0592	0.0929
Bifidobacterium_387352	RUG472	-0.4421	0.0581	0.0919
Bifidobacterium_388775	RUG472	-0.5140	0.0244	0.0543
CAG-177	RUG472	0.4614	0.0468	0.0808
Desulfovibrio_R_446353	RUG472	0.5281	0.0201	0.0466
Galliscardovia_388776	RUG472	-0.5002	0.0292	0.0609
HUN007	RUG472	-0.4958	0.0309	0.0630
RUG11690	RUG472	-0.0105	0.9659	0.9659
Shoulder IMF	Stomatobaculum	-0.6636	0.0027	0.0152
Rump IMF	Stomatobaculum	-0.2123	0.3829	0.4206

Var1	Var2	Spearman_rho	P_value	FDR
LDL（mmol/L）	Stomatobaculum	-0.5561	0.0134	0.0392
HDL（mmol/L）	Stomatobaculum	-0.5344	0.0184	0.0460
VLDL（mmol/L）	Stomatobaculum	-0.3672	0.1220	0.1588
FFA（mmol/L）	Stomatobaculum	-0.2228	0.3592	0.3991
TG（mmol/L）	Stomatobaculum	-0.5123	0.0249	0.0544
TC（mmol/L）	Stomatobaculum	-0.4632	0.0458	0.0803
Bifidobacterium_387352	Stomatobaculum	0.4158	0.0766	0.1120
Bifidobacterium_388775	Stomatobaculum	0.6491	0.0026	0.0152
CAG-177	Stomatobaculum	-0.4649	0.0449	0.0797
Desulfovibrio_R_446353	Stomatobaculum	-0.5439	0.0161	0.0430
Galliscardovia_388776	Stomatobaculum	0.5459	0.0156	0.0426
HUN007	Stomatobaculum	0.6907	0.0011	0.0088
RUG11690	Stomatobaculum	-0.1088	0.6576	0.6903
RUG472	Stomatobaculum	-0.3456	0.1472	0.1829
Shoulder IMF	UBA1394	-0.4035	0.0968	0.1343
Rump IMF	UBA1394	-0.4421	0.0581	0.0919
LDL（mmol/L）	UBA1394	-0.4246	0.0700	0.1039
HDL（mmol/L）	UBA1394	-0.3370	0.1583	0.1940
VLDL（mmol/L）	UBA1394	-0.3144	0.1898	0.2268
FFA（mmol/L）	UBA1394	-0.3439	0.1494	0.1844
TG（mmol/L）	UBA1394	-0.2298	0.3439	0.3889
TC（mmol/L）	UBA1394	-0.1860	0.4459	0.4841
Bifidobacterium_387352	UBA1394	0.2246	0.3553	0.3972
Bifidobacterium_388775	UBA1394	0.3807	0.1078	0.1453
CAG-177	UBA1394	-0.4509	0.0527	0.0878
Desulfovibrio_R_446353	UBA1394	-0.4930	0.0320	0.0633
Galliscardovia_388776	UBA1394	0.3853	0.1033	0.1413
HUN007	UBA1394	0.5774	0.0096	0.0349
RUG11690	UBA1394	0.1596	0.5138	0.5485
RUG472	UBA1394	-0.6632	0.0020	0.0134
Stomatobaculum	UBA1394	0.4281	0.0675	0.1026
Shoulder IMF	Ventrimonas	-0.6649	0.0026	0.0152
Rump IMF	Ventrimonas	-0.1387	0.5713	0.6064
LDL（mmol/L）	Ventrimonas	-0.4520	0.0521	0.0875
HDL（mmol/L）	Ventrimonas	-0.5285	0.0200	0.0466
VLDL（mmol/L）	Ventrimonas	-0.2513	0.2993	0.3447
FFA（mmol/L）	Ventrimonas	-0.2194	0.3668	0.4052
TG（mmol/L）	Ventrimonas	-0.5134	0.0246	0.0543
TC（mmol/L）	Ventrimonas	-0.3458	0.1471	0.1829
Bifidobacterium_387352	Ventrimonas	0.3589	0.1313	0.1674
Bifidobacterium_388775	Ventrimonas	0.5371	0.0177	0.0455
CAG-177	Ventrimonas	-0.6433	0.0030	0.0152

Var1	Var2	Spearman_rho	P_value	FDR
Desulfovibrio_R_446353	Ventrimonas	-0.4581	0.0486	0.0824
Galliscardovia_388776	Ventrimonas	0.4320	0.0648	0.1000
HUN007	Ventrimonas	0.6277	0.0040	0.0190
RUG11690	Ventrimonas	-0.1264	0.6062	0.6399
RUG472	Ventrimonas	-0.4484	0.0541	0.0887
Stomatobaculum	Ventrimonas	0.7740	0.0001	0.0019
UBA1394	Ventrimonas	0.5713	0.0106	0.0364
