# Supplementary material for: Gut Microbiome Signatures of Aging Associated with Intramuscular Fat Deposition in Tan Sheep
Source: Animals (Basel). 2026 Feb 19;16(4):661. doi: 10.3390/ani16040661 (PMC12937419; doi:10.3390/ani16040661)
Supplement: Supplementary file 1 [file animals-16-00661-s001.zip › Supplementary Table S5c.pdf]

**Supplementary Table S5c**

Correlations between specific gut bacterial taxa and IMF deposition indicators (In colon)

| Var1 | Var2 | Spearman_rho | P_value | FDR |
|---|---|---|---|---|
| Shoulder meat | Rump meat | 0.4772 | 0.0388 | 0.0895 |
| Shoulder meat | LDL（mmol/L） | 0.7647 | 0.0002 | 0.0036 |
| Rump meat | LDL（mmol/L） | 0.4947 | 0.0313 | 0.0770 |
| Shoulder meat | HDL（mmol/L） | 0.7888 | 0.0001 | 0.0027 |
| Rump meat | HDL（mmol/L） | 0.3124 | 0.1928 | 0.2579 |
| LDL（mmol/L） | HDL（mmol/L） | 0.6845 | 0.0012 | 0.0133 |
| Shoulder meat | VLDL（mmol/L） | 0.7355 | 0.0005 | 0.0074 |
| Rump meat | VLDL（mmol/L） | 0.5815 | 0.0090 | 0.0407 |
| LDL（mmol/L） | VLDL（mmol/L） | 0.6465 | 0.0028 | 0.0201 |
| HDL（mmol/L） | VLDL（mmol/L） | 0.7179 | 0.0005 | 0.0076 |
| Shoulder meat | FFA（mmol/L） | 0.5459 | 0.0191 | 0.0592 |
| Rump meat | FFA（mmol/L） | 0.4842 | 0.0357 | 0.0852 |
| LDL（mmol/L） | FFA（mmol/L） | 0.8684 | 0.0000 | 0.0002 |
| HDL（mmol/L） | FFA（mmol/L） | 0.6757 | 0.0015 | 0.0150 |
| VLDL（mmol/L） | FFA（mmol/L） | 0.6105 | 0.0055 | 0.0308 |
| Shoulder meat | TG（mmol/L） | 0.8019 | 0.0001 | 0.0021 |
| Rump meat | TG（mmol/L） | 0.3965 | 0.0928 | 0.1479 |
| LDL（mmol/L） | TG（mmol/L） | 0.7614 | 0.0002 | 0.0030 |
| HDL（mmol/L） | TG（mmol/L） | 0.7995 | 0.0000 | 0.0021 |
| VLDL（mmol/L） | TG（mmol/L） | 0.7589 | 0.0002 | 0.0030 |
| FFA（mmol/L） | TG（mmol/L） | 0.6000 | 0.0066 | 0.0341 |
| Shoulder meat | TC（mmol/L） | 0.6594 | 0.0029 | 0.0201 |
| Rump meat | TC（mmol/L） | 0.4246 | 0.0700 | 0.1273 |
| LDL（mmol/L） | TC（mmol/L） | 0.7947 | 0.0000 | 0.0021 |
| HDL（mmol/L） | TC（mmol/L） | 0.7328 | 0.0004 | 0.0056 |
| VLDL（mmol/L） | TC（mmol/L） | 0.7668 | 0.0001 | 0.0030 |
| FFA（mmol/L） | TC（mmol/L） | 0.7596 | 0.0002 | 0.0030 |
| TG（mmol/L） | TC（mmol/L） | 0.7877 | 0.0001 | 0.0021 |
| Shoulder meat | Acetate（ug/g） | -0.6941 | 0.0029 | 0.0201 |
| Rump meat | Acetate（ug/g） | -0.2735 | 0.3053 | 0.3662 |
| LDL（mmol/L） | Acetate（ug/g） | -0.7176 | 0.0017 | 0.0162 |
| HDL（mmol/L） | Acetate（ug/g） | -0.7991 | 0.0002 | 0.0035 |
| VLDL（mmol/L） | Acetate（ug/g） | -0.6176 | 0.0108 | 0.0433 |
| FFA（mmol/L） | Acetate（ug/g） | -0.6647 | 0.0050 | 0.0294 |
| TG（mmol/L） | Acetate（ug/g） | -0.6794 | 0.0038 | 0.0247 |
| TC（mmol/L） | Acetate（ug/g） | -0.6647 | 0.0050 | 0.0294 |
| Shoulder meat | Propionate（ug/g） | -0.6971 | 0.0027 | 0.0201 |
| Rump meat | Propionate（ug/g） | -0.4382 | 0.0895 | 0.1448 |

| Var1 | Var2 | Spearman_rho | P_value | FDR |
|---|---|---|---|---|
| LDL（mmol/L） | Propionate（ug/g） | -0.8088 | 0.0001 | 0.0030 |
| HDL（mmol/L） | Propionate（ug/g） | -0.7403 | 0.0010 | 0.0117 |
| VLDL（mmol/L） | Propionate（ug/g） | -0.6000 | 0.0140 | 0.0489 |
| FFA（mmol/L） | Propionate（ug/g） | -0.7000 | 0.0025 | 0.0201 |
| TG（mmol/L） | Propionate（ug/g） | -0.6941 | 0.0029 | 0.0201 |
| TC（mmol/L） | Propionate（ug/g） | -0.7529 | 0.0008 | 0.0095 |
| Acetate（ug/g） | Propionate（ug/g） | 0.9235 | 0.0000 | 0.0001 |
| Shoulder meat | Butyrate（ug/g） | -0.6265 | 0.0094 | 0.0413 |
| Rump meat | Butyrate（ug/g） | -0.5059 | 0.0456 | 0.0981 |
| LDL（mmol/L） | Butyrate（ug/g） | -0.6471 | 0.0067 | 0.0342 |
| HDL（mmol/L） | Butyrate（ug/g） | -0.6225 | 0.0100 | 0.0425 |
| VLDL（mmol/L） | Butyrate（ug/g） | -0.6265 | 0.0094 | 0.0413 |
| FFA（mmol/L） | Butyrate（ug/g） | -0.6059 | 0.0129 | 0.0482 |
| TG（mmol/L） | Butyrate（ug/g） | -0.5118 | 0.0427 | 0.0953 |
| TC（mmol/L） | Butyrate（ug/g） | -0.6088 | 0.0123 | 0.0471 |
| Acetate（ug/g） | Butyrate（ug/g） | 0.8294 | 0.0001 | 0.0021 |
| Propionate（ug/g） | Butyrate（ug/g） | 0.8382 | 0.0001 | 0.0021 |
| Shoulder meat | CAG-269 | -0.4489 | 0.0617 | 0.1186 |
| Rump meat | CAG-269 | -0.4228 | 0.0713 | 0.1287 |
| LDL（mmol/L） | CAG-269 | -0.4825 | 0.0364 | 0.0858 |
| HDL（mmol/L） | CAG-269 | -0.7100 | 0.0007 | 0.0086 |
| VLDL（mmol/L） | CAG-269 | -0.3285 | 0.1697 | 0.2355 |
| FFA（mmol/L） | CAG-269 | -0.5158 | 0.0238 | 0.0672 |
| TG（mmol/L） | CAG-269 | -0.4193 | 0.0739 | 0.1306 |
| TC（mmol/L） | CAG-269 | -0.4263 | 0.0687 | 0.1269 |
| Acetate（ug/g） | CAG-269 | 0.4529 | 0.0781 | 0.1350 |
| Propionate（ug/g） | CAG-269 | 0.5412 | 0.0304 | 0.0769 |
| Butyrate（ug/g） | CAG-269 | 0.4088 | 0.1159 | 0.1802 |
| Shoulder meat | Copromorpha | 0.5624 | 0.0151 | 0.0510 |
| Rump meat | Copromorpha | 0.5368 | 0.0178 | 0.0572 |
| LDL（mmol/L） | Copromorpha | 0.6912 | 0.0010 | 0.0117 |
| HDL（mmol/L） | Copromorpha | 0.4642 | 0.0452 | 0.0980 |
| VLDL（mmol/L） | Copromorpha | 0.5832 | 0.0088 | 0.0407 |
| FFA（mmol/L） | Copromorpha | 0.5561 | 0.0134 | 0.0484 |
| TG（mmol/L） | Copromorpha | 0.5737 | 0.0102 | 0.0425 |
| TC（mmol/L） | Copromorpha | 0.6737 | 0.0016 | 0.0150 |
| Acetate（ug/g） | Copromorpha | -0.5941 | 0.0152 | 0.0510 |
| Propionate（ug/g） | Copromorpha | -0.8294 | 0.0001 | 0.0021 |
| Butyrate（ug/g） | Copromorpha | -0.6206 | 0.0103 | 0.0425 |
| CAG-269 | Copromorpha | -0.4333 | 0.0638 | 0.1208 |
| Shoulder meat | Cryptobacteroides | -0.6491 | 0.0036 | 0.0236 |
| Rump meat | Cryptobacteroides | -0.3158 | 0.1878 | 0.2522 |

| Var1 | Var2 | Spearman_rho | P_value | FDR |
|---|---|---|---|---|
| LDL（mmol/L） | Cryptobacteroides | -0.5123 | 0.0249 | 0.0687 |
| HDL（mmol/L） | Cryptobacteroides | -0.5713 | 0.0106 | 0.0431 |
| VLDL（mmol/L） | Cryptobacteroides | -0.6632 | 0.0020 | 0.0173 |
| FFA（mmol/L） | Cryptobacteroides | -0.3877 | 0.1010 | 0.1593 |
| TG（mmol/L） | Cryptobacteroides | -0.4965 | 0.0306 | 0.0769 |
| TC（mmol/L） | Cryptobacteroides | -0.4439 | 0.0570 | 0.1143 |
| Acetate（ug/g） | Cryptobacteroides | 0.6588 | 0.0055 | 0.0308 |
| Propionate（ug/g） | Cryptobacteroides | 0.6735 | 0.0042 | 0.0265 |
| Butyrate（ug/g） | Cryptobacteroides | 0.4382 | 0.0895 | 0.1448 |
| CAG-269 | Cryptobacteroides | 0.2596 | 0.2830 | 0.3458 |
| Copromorpha | Cryptobacteroides | -0.5912 | 0.0077 | 0.0372 |
| Shoulder meat | DTU089 | 0.4943 | 0.0370 | 0.0864 |
| Rump meat | DTU089 | 0.2263 | 0.3515 | 0.4051 |
| LDL（mmol/L） | DTU089 | 0.4175 | 0.0753 | 0.1322 |
| HDL（mmol/L） | DTU089 | 0.4212 | 0.0725 | 0.1297 |
| VLDL（mmol/L） | DTU089 | 0.4014 | 0.0885 | 0.1445 |
| FFA（mmol/L） | DTU089 | 0.1947 | 0.4243 | 0.4756 |
| TG（mmol/L） | DTU089 | 0.3491 | 0.1429 | 0.2102 |
| TC（mmol/L） | DTU089 | 0.5053 | 0.0273 | 0.0728 |
| Acetate（ug/g） | DTU089 | -0.4559 | 0.0759 | 0.1327 |
| Propionate（ug/g） | DTU089 | -0.5765 | 0.0194 | 0.0592 |
| Butyrate（ug/g） | DTU089 | -0.2941 | 0.2688 | 0.3335 |
| CAG-269 | DTU089 | -0.3632 | 0.1265 | 0.1939 |
| Copromorpha | DTU089 | 0.5965 | 0.0070 | 0.0349 |
| Cryptobacteroides | DTU089 | -0.5877 | 0.0081 | 0.0389 |
| Shoulder meat | Mobilitalea | 0.2797 | 0.2610 | 0.3250 |
| Rump meat | Mobilitalea | 0.3509 | 0.1408 | 0.2089 |
| LDL（mmol/L） | Mobilitalea | 0.4596 | 0.0477 | 0.1020 |
| HDL（mmol/L） | Mobilitalea | 0.4520 | 0.0521 | 0.1078 |
| VLDL（mmol/L） | Mobilitalea | 0.5077 | 0.0265 | 0.0719 |
| FFA（mmol/L） | Mobilitalea | 0.5684 | 0.0111 | 0.0435 |
| TG（mmol/L） | Mobilitalea | 0.4474 | 0.0548 | 0.1106 |
| TC（mmol/L） | Mobilitalea | 0.3509 | 0.1408 | 0.2089 |
| Acetate（ug/g） | Mobilitalea | -0.3382 | 0.2001 | 0.2645 |
| Propionate（ug/g） | Mobilitalea | -0.2765 | 0.2999 | 0.3610 |
| Butyrate（ug/g） | Mobilitalea | -0.2265 | 0.3990 | 0.4518 |
| CAG-269 | Mobilitalea | -0.3368 | 0.1585 | 0.2259 |
| Copromorpha | Mobilitalea | 0.2298 | 0.3439 | 0.4006 |
| Cryptobacteroides | Mobilitalea | -0.2807 | 0.2444 | 0.3103 |
| DTU089 | Mobilitalea | 0.0386 | 0.8753 | 0.8808 |
| Shoulder meat | Onthenecus | 0.4180 | 0.0844 | 0.1406 |
| Rump meat | Onthenecus | 0.4333 | 0.0638 | 0.1208 |

| Var1 | Var2 | Spearman_rho | P_value | FDR |
|---|---|---|---|---|
| LDL（mmol/L） | Onthenecus | 0.4158 | 0.0766 | 0.1332 |
| HDL（mmol/L） | Onthenecus | 0.4037 | 0.0865 | 0.1423 |
| VLDL（mmol/L） | Onthenecus | 0.2749 | 0.2546 | 0.3208 |
| FFA（mmol/L） | Onthenecus | 0.3754 | 0.1132 | 0.1769 |
| TG（mmol/L） | Onthenecus | 0.2807 | 0.2444 | 0.3103 |
| TC（mmol/L） | Onthenecus | 0.3158 | 0.1878 | 0.2522 |
| Acetate（ug/g） | Onthenecus | -0.6559 | 0.0058 | 0.0314 |
| Propionate（ug/g） | Onthenecus | -0.7235 | 0.0015 | 0.0150 |
| Butyrate（ug/g） | Onthenecus | -0.5265 | 0.0362 | 0.0858 |
| CAG-269 | Onthenecus | -0.4105 | 0.0808 | 0.1375 |
| Copromorpha | Onthenecus | 0.6667 | 0.0018 | 0.0165 |
| Cryptobacteroides | Onthenecus | -0.4965 | 0.0306 | 0.0769 |
| DTU089 | Onthenecus | 0.6175 | 0.0048 | 0.0294 |
| Mobilitalea | Onthenecus | 0.1772 | 0.4680 | 0.5104 |
| Shoulder meat | PeH17 | -0.5501 | 0.0180 | 0.0574 |
| Rump meat | PeH17 | -0.3421 | 0.1517 | 0.2191 |
| LDL（mmol/L） | PeH17 | -0.4947 | 0.0313 | 0.0770 |
| HDL（mmol/L） | PeH17 | -0.3273 | 0.1713 | 0.2359 |
| VLDL（mmol/L） | PeH17 | -0.4383 | 0.0605 | 0.1178 |
| FFA（mmol/L） | PeH17 | -0.2316 | 0.3401 | 0.3990 |
| TG（mmol/L） | PeH17 | -0.4421 | 0.0581 | 0.1151 |
| TC（mmol/L） | PeH17 | -0.2772 | 0.2506 | 0.3169 |
| Acetate（ug/g） | PeH17 | 0.6441 | 0.0071 | 0.0349 |
| Propionate（ug/g） | PeH17 | 0.7235 | 0.0015 | 0.0150 |
| Butyrate（ug/g） | PeH17 | 0.5471 | 0.0283 | 0.0748 |
| CAG-269 | PeH17 | 0.1456 | 0.5520 | 0.5920 |
| Copromorpha | PeH17 | -0.7018 | 0.0008 | 0.0098 |
| Cryptobacteroides | PeH17 | 0.7947 | 0.0000 | 0.0021 |
| DTU089 | PeH17 | -0.5298 | 0.0196 | 0.0592 |
| Mobilitalea | PeH17 | -0.1018 | 0.6785 | 0.7023 |
| Onthenecus | PeH17 | -0.6596 | 0.0021 | 0.0181 |
| Shoulder meat | RUG420 | 0.5377 | 0.0214 | 0.0622 |
| Rump meat | RUG420 | 0.5000 | 0.0293 | 0.0767 |
| LDL（mmol/L） | RUG420 | 0.4684 | 0.0431 | 0.0953 |
| HDL（mmol/L） | RUG420 | 0.5845 | 0.0086 | 0.0405 |
| VLDL（mmol/L） | RUG420 | 0.5279 | 0.0202 | 0.0596 |
| FFA（mmol/L） | RUG420 | 0.4982 | 0.0299 | 0.0769 |
| TG（mmol/L） | RUG420 | 0.4246 | 0.0700 | 0.1273 |
| TC（mmol/L） | RUG420 | 0.4404 | 0.0592 | 0.1166 |
| Acetate（ug/g） | RUG420 | -0.4294 | 0.0969 | 0.1537 |
| Propionate（ug/g） | RUG420 | -0.5412 | 0.0304 | 0.0769 |
| Butyrate（ug/g） | RUG420 | -0.4794 | 0.0602 | 0.1178 |

| Var1 | Var2 | Spearman_rho | P_value | FDR |
|---|---|---|---|---|
| CAG-269 | RUG420 | -0.7982 | 0.0000 | 0.0021 |
| Copromorpha | RUG420 | 0.6053 | 0.0060 | 0.0321 |
| Cryptobacteroides | RUG420 | -0.4211 | 0.0726 | 0.1297 |
| DTU089 | RUG420 | 0.3298 | 0.1679 | 0.2352 |
| Mobilitalea | RUG420 | 0.3439 | 0.1494 | 0.2178 |
| Onthenecus | RUG420 | 0.3333 | 0.1631 | 0.2305 |
| PeH17 | RUG420 | -0.3333 | 0.1631 | 0.2305 |
| Shoulder meat | Treponema_C | -0.5645 | 0.0147 | 0.0502 |
| Rump meat | Treponema_C | -0.2246 | 0.3553 | 0.4081 |
| LDL（mmol/L） | Treponema_C | -0.4526 | 0.0517 | 0.1076 |
| HDL（mmol/L） | Treponema_C | -0.3967 | 0.0927 | 0.1479 |
| VLDL（mmol/L） | Treponema_C | -0.2407 | 0.3209 | 0.3807 |
| FFA（mmol/L） | Treponema_C | -0.2526 | 0.2967 | 0.3585 |
| TG（mmol/L） | Treponema_C | -0.3404 | 0.1539 | 0.2204 |
| TC（mmol/L） | Treponema_C | -0.3667 | 0.1226 | 0.1897 |
| Acetate（ug/g） | Treponema_C | 0.3559 | 0.1761 | 0.2395 |
| Propionate（ug/g） | Treponema_C | 0.6000 | 0.0140 | 0.0489 |
| Butyrate（ug/g） | Treponema_C | 0.2882 | 0.2790 | 0.3422 |
| CAG-269 | Treponema_C | 0.4088 | 0.0823 | 0.1390 |
| Copromorpha | Treponema_C | -0.6579 | 0.0022 | 0.0183 |
| Cryptobacteroides | Treponema_C | 0.5509 | 0.0145 | 0.0502 |
| DTU089 | Treponema_C | -0.6035 | 0.0062 | 0.0326 |
| Mobilitalea | Treponema_C | 0.1702 | 0.4861 | 0.5284 |
| Onthenecus | Treponema_C | -0.4526 | 0.0517 | 0.1076 |
| PeH17 | Treponema_C | 0.4895 | 0.0334 | 0.0810 |
| RUG420 | Treponema_C | -0.4105 | 0.0808 | 0.1375 |
| Shoulder meat | UBA1067 | 0.3168 | 0.2002 | 0.2645 |
| Rump meat | UBA1067 | 0.1930 | 0.4286 | 0.4787 |
| LDL（mmol/L） | UBA1067 | 0.3018 | 0.2093 | 0.2699 |
| HDL（mmol/L） | UBA1067 | 0.5731 | 0.0103 | 0.0425 |
| VLDL（mmol/L） | UBA1067 | 0.2934 | 0.2228 | 0.2851 |
| FFA（mmol/L） | UBA1067 | 0.3298 | 0.1679 | 0.2352 |
| TG（mmol/L） | UBA1067 | 0.3035 | 0.2065 | 0.2695 |
| TC（mmol/L） | UBA1067 | 0.2368 | 0.3289 | 0.3887 |
| Acetate（ug/g） | UBA1067 | -0.1735 | 0.5204 | 0.5638 |
| Propionate（ug/g） | UBA1067 | -0.1706 | 0.5276 | 0.5697 |
| Butyrate（ug/g） | UBA1067 | -0.2324 | 0.3865 | 0.4408 |
| CAG-269 | UBA1067 | -0.6561 | 0.0023 | 0.0185 |
| Copromorpha | UBA1067 | 0.0965 | 0.6943 | 0.7141 |
| Cryptobacteroides | UBA1067 | -0.3281 | 0.1703 | 0.2355 |
| DTU089 | UBA1067 | 0.1298 | 0.5963 | 0.6313 |
| Mobilitalea | UBA1067 | 0.3491 | 0.1429 | 0.2102 |

| Var1 | Var2 | Spearman_rho | P_value | FDR |
|---|---|---|---|---|
| Onthenecus | UBA1067 | 0.0316 | 0.8979 | 0.9007 |
| PeH17 | UBA1067 | -0.1228 | 0.6165 | 0.6505 |
| RUG420 | UBA1067 | 0.4842 | 0.0357 | 0.0852 |
| Treponema_C | UBA1067 | -0.1035 | 0.6733 | 0.6991 |
| Shoulder meat | UBA5905 | 0.5913 | 0.0097 | 0.0422 |
| Rump meat | UBA5905 | 0.4193 | 0.0739 | 0.1306 |
| LDL（mmol/L） | UBA5905 | 0.5298 | 0.0196 | 0.0592 |
| HDL（mmol/L） | UBA5905 | 0.6450 | 0.0029 | 0.0201 |
| VLDL（mmol/L） | UBA5905 | 0.6096 | 0.0056 | 0.0308 |
| FFA（mmol/L） | UBA5905 | 0.4509 | 0.0527 | 0.1084 |
| TG（mmol/L） | UBA5905 | 0.5368 | 0.0178 | 0.0572 |
| TC（mmol/L） | UBA5905 | 0.5228 | 0.0216 | 0.0622 |
| Acetate（ug/g） | UBA5905 | -0.5088 | 0.0441 | 0.0969 |
| Propionate（ug/g） | UBA5905 | -0.6000 | 0.0140 | 0.0489 |
| Butyrate（ug/g） | UBA5905 | -0.5676 | 0.0218 | 0.0622 |
| CAG-269 | UBA5905 | -0.7105 | 0.0007 | 0.0086 |
| Copromorpha | UBA5905 | 0.5228 | 0.0216 | 0.0622 |
| Cryptobacteroides | UBA5905 | -0.3281 | 0.1703 | 0.2355 |
| DTU089 | UBA5905 | 0.4649 | 0.0449 | 0.0979 |
| Mobilitalea | UBA5905 | 0.2649 | 0.2731 | 0.3361 |
| Onthenecus | UBA5905 | 0.2333 | 0.3364 | 0.3961 |
| PeH17 | UBA5905 | -0.2281 | 0.3477 | 0.4036 |
| RUG420 | UBA5905 | 0.7684 | 0.0001 | 0.0030 |
| Treponema_C | UBA5905 | -0.4561 | 0.0497 | 0.1048 |
| UBA1067 | UBA5905 | 0.4035 | 0.0867 | 0.1423 |
| Shoulder meat | UBA737 | -0.3767 | 0.1234 | 0.1900 |
| Rump meat | UBA737 | 0.0947 | 0.6997 | 0.7173 |
| LDL（mmol/L） | UBA737 | -0.3404 | 0.1539 | 0.2204 |
| HDL（mmol/L） | UBA737 | -0.5581 | 0.0130 | 0.0482 |
| VLDL（mmol/L） | UBA737 | -0.5296 | 0.0197 | 0.0592 |
| FFA（mmol/L） | UBA737 | -0.4281 | 0.0675 | 0.1261 |
| TG（mmol/L） | UBA737 | -0.4421 | 0.0581 | 0.1151 |
| TC（mmol/L） | UBA737 | -0.4070 | 0.0837 | 0.1403 |
| Acetate（ug/g） | UBA737 | 0.6588 | 0.0055 | 0.0308 |
| Propionate（ug/g） | UBA737 | 0.5147 | 0.0413 | 0.0940 |
| Butyrate（ug/g） | UBA737 | 0.3941 | 0.1309 | 0.1997 |
| CAG-269 | UBA737 | 0.2035 | 0.4034 | 0.4552 |
| Copromorpha | UBA737 | -0.3070 | 0.2011 | 0.2645 |
| Cryptobacteroides | UBA737 | 0.4807 | 0.0372 | 0.0864 |
| DTU089 | UBA737 | -0.1912 | 0.4329 | 0.4818 |
| Mobilitalea | UBA737 | -0.1860 | 0.4459 | 0.4929 |
| Onthenecus | UBA737 | -0.2263 | 0.3515 | 0.4051 |

| Var1 | Var2 | Spearman_rho | P_value | FDR |
|---|---|---|---|---|
| PeH17 | UBA737 | 0.3544 | 0.1366 | 0.2055 |
| RUG420 | UBA737 | -0.3246 | 0.1752 | 0.2392 |
| Treponema_C | UBA737 | 0.1140 | 0.6420 | 0.6709 |
| UBA1067 | UBA737 | -0.1825 | 0.4547 | 0.4976 |
| UBA5905 | UBA737 | -0.4105 | 0.0808 | 0.1375 |
| Shoulder meat | UMGS1994 | -0.2528 | 0.3114 | 0.3707 |
| Rump meat | UMGS1994 | -0.1035 | 0.6733 | 0.6991 |
| LDL（mmol/L） | UMGS1994 | -0.4754 | 0.0397 | 0.0908 |
| HDL（mmol/L） | UMGS1994 | -0.5344 | 0.0184 | 0.0581 |
| VLDL（mmol/L） | UMGS1994 | -0.2073 | 0.3945 | 0.4483 |
| FFA（mmol/L） | UMGS1994 | -0.5368 | 0.0178 | 0.0572 |
| TG（mmol/L） | UMGS1994 | -0.4491 | 0.0537 | 0.1091 |
| TC（mmol/L） | UMGS1994 | -0.4281 | 0.0675 | 0.1261 |
| Acetate（ug/g） | UMGS1994 | 0.6147 | 0.0113 | 0.0436 |
| Propionate（ug/g） | UMGS1994 | 0.4912 | 0.0534 | 0.1090 |
| Butyrate（ug/g） | UMGS1994 | 0.2059 | 0.4443 | 0.4928 |
| CAG-269 | UMGS1994 | 0.4368 | 0.0615 | 0.1186 |
| Copromorpha | UMGS1994 | -0.2298 | 0.3439 | 0.4006 |
| Cryptobacteroides | UMGS1994 | 0.2649 | 0.2731 | 0.3361 |
| DTU089 | UMGS1994 | -0.2526 | 0.2967 | 0.3585 |
| Mobilitalea | UMGS1994 | -0.5579 | 0.0131 | 0.0482 |
| Onthenecus | UMGS1994 | -0.4579 | 0.0487 | 0.1034 |
| PeH17 | UMGS1994 | 0.1965 | 0.4201 | 0.4724 |
| RUG420 | UMGS1994 | -0.2456 | 0.3108 | 0.3707 |
| Treponema_C | UMGS1994 | -0.0070 | 0.9773 | 0.9773 |
| UBA1067 | UMGS1994 | -0.1456 | 0.5520 | 0.5920 |
| UBA5905 | UMGS1994 | -0.0544 | 0.8250 | 0.8353 |
| UBA737 | UMGS1994 | 0.2719 | 0.2601 | 0.3250 |
| Shoulder meat | Vescimonas | 0.4365 | 0.0701 | 0.1273 |
| Rump meat | Vescimonas | 0.2982 | 0.2149 | 0.2760 |
| LDL（mmol/L） | Vescimonas | 0.0825 | 0.7372 | 0.7534 |
| HDL（mmol/L） | Vescimonas | 0.5125 | 0.0249 | 0.0687 |
| VLDL（mmol/L） | Vescimonas | 0.3469 | 0.1456 | 0.2131 |
| FFA（mmol/L） | Vescimonas | 0.0965 | 0.6943 | 0.7141 |
| TG（mmol/L） | Vescimonas | 0.3544 | 0.1366 | 0.2055 |
| TC（mmol/L） | Vescimonas | 0.1298 | 0.5963 | 0.6313 |
| Acetate（ug/g） | Vescimonas | -0.5853 | 0.0172 | 0.0571 |
| Propionate（ug/g） | Vescimonas | -0.4471 | 0.0825 | 0.1390 |
| Butyrate（ug/g） | Vescimonas | -0.4735 | 0.0639 | 0.1208 |
| CAG-269 | Vescimonas | -0.3526 | 0.1387 | 0.2077 |
| Copromorpha | Vescimonas | 0.1351 | 0.5814 | 0.6195 |
| Cryptobacteroides | Vescimonas | -0.3018 | 0.2093 | 0.2699 |

| Var1 | Var2 | Spearman_rho | P_value | FDR |
|---|---|---|---|---|
| DTU089 | Vescimonas | 0.1825 | 0.4547 | 0.4976 |
| Mobilitalea | Vescimonas | 0.1211 | 0.6215 | 0.6537 |
| Onthenecus | Vescimonas | 0.4702 | 0.0422 | 0.0953 |
| PeH17 | Vescimonas | -0.3018 | 0.2093 | 0.2699 |
| RUG420 | Vescimonas | 0.3561 | 0.1345 | 0.2043 |
| Treponema_C | Vescimonas | -0.1140 | 0.6420 | 0.6709 |
| UBA1067 | Vescimonas | 0.0439 | 0.8585 | 0.8665 |
| UBA5905 | Vescimonas | 0.3193 | 0.1827 | 0.2474 |
| UBA737 | Vescimonas | -0.2544 | 0.2933 | 0.3570 |
| UMGS1994 | Vescimonas | -0.3263 | 0.1727 | 0.2369 |
